# Reshaping Tumor-Lymph Node Immune Axis via Targeted Lymphatic Delivery of Dual-Functional Immune Modulator for Enhanced Cancer Immunotherapy

**DOI:** 10.1021/acscentsci.5c00509

**Published:** 2025-09-17

**Authors:** Su Yeon Lim, Pin Liu, Ju Hwa Shin, Bum Soo Lee, Sun Ju Kim, Dahwun Kim, Siyan Lyu, Byung Deok Kim, Chaeeun Park, Junku Jung, Jihyun Lee, Jinbeom Seo, Taegwan Yun, Hyo Jin Park, Min Sang Lee, Ki Hyun Kim, Wonsik Lee, Ji Hoon Jeong

**Affiliations:** † School of Pharmacy, 105934 Sungkyunkwan University, Suwon 16419, Republic of Korea; ‡ Department of MetaBioHealth, Institute for Cross-disciplinary Studies, Sungkyunkwan University, Suwon 16419, Republic of Korea; § Biomedical Institute for Convergence at SKKU, Sungkyunkwan University, Suwon 16419, Republic of Korea; # Gyeonggido Business and Science Accelerator, Suwon-si, Gyeonggi-do 16229, Republic of Korea

## Abstract

The efficacy of cancer immunotherapy is often limited by the immunosuppressive tumor microenvironment (TME) and insufficient immune activation in tumor-draining lymph nodes (TDLN). Since the TME and TDLN form a dynamic axis crucial for tumor metastasis and resistance to immune checkpoint blockade, strategies that effectively modulate both sites are critical. Here, we present a dissolving microneedle (MN) system that generates nanomicelles (NMCs) for localized delivery of a newly identified dual-functional macrocyclic trichothecene, Roridin E (R.E). R.E induces cancer cell-autonomous secretion of IFN-β and immunogenic cancer cell death (ICD). Direct delivery of R.E to the TDLN via the MN platform reshapes the local immune landscape to suppress cancer while minimizing off-target toxicity. In a B16F10 melanoma model, MN-guided R.E. delivery significantly improved tumor control, reduced lung metastases, and extended overall survival. This approach provides a minimally invasive and effective strategy for integrating natural-product-based therapies with advanced drug delivery systems to target the TME–TDLN axis, thereby improving outcomes in metastatic cancer.

## Graphical abstract

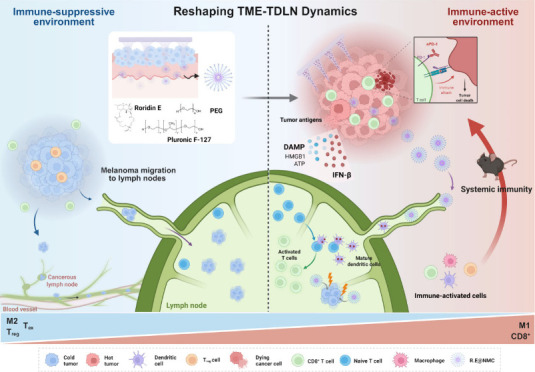

## Introduction

Previous studies have emphasized that the key players in antitumor immunity reside in both the tumor microenvironment (TME) and the draining lymph nodes (TDLN), suggesting that effective immunotherapies should address both sites. The lymphatic system, particularly TDLN, plays a pivotal role in the early stages of metastasis and in shaping the TME.^1^ The dynamic interplay between the TME and TDLN forms a critical axis, not only facilitating immune evasion by the tumor but also serving as a conduit for the dissemination of cancer cells to distant organs. TDLN acts as primary immunological sites for tumor antigen recognition, initially enabling immune activation.^2,3^ However, as tumors progress, TDLN often transitions into permissive environments that promote immune suppression and facilitate metastatic spread.^3,4^

Immune checkpoint inhibitors (ICIs) have revolutionized cancer treatment by unleashing anticancer immune responses, primarily mediated by CD8^+^ T cells.^5^ Despite these advances, many patients exhibit intrinsic or acquired resistance to ICIs.^6^ This resistance is partly attributed to the immunosuppressive microenvironment within metastatic TDLN,^7^ where systemic administration of ICIs often fails due to poor drug accumulation and retention.^8,9^ Furthermore, the immunosuppressive milieu in TME and TDLN promotes immune tolerance, reduces the efficacy of ICIs, and consequently compromises the effector cell function in tumor,^10^ suggesting the need for the simultaneous control of immunity in both TME and TDLN.^8,11^

Although the systemic delivery of immune modulators may be considered as an option for the therapeutic intervention, this can cause severe side effects and often fails to concentrate sufficient activity in the TDLN, leading to suboptimal T-cell priming.^12^ To address these challenges, the locoregional delivery of an immune modulator targeting the TME–TDLN axis may offer a promising alternative. In this study, we explored the therapeutic potential of roridin E (R.E), a macrocyclic trichothecene mycotoxin derived from the poisonous mushroom *Podostroma cornu-damae*. For the first time, we found that R.E has a dual action to induce immunogenic cell death (ICD) and stimulates secretion of interferon β (IFN-β) in cancer cells. These unique responses may promote anticancer immunity through diverse mechanisms,^13^ positioning R.E as a promising candidate for cancer immunotherapy.

To minimize potential off-target toxicity and systemic immune-related adverse effects of R.E, we hypothesized that targeting the TME–TDLN axis via the MN-directed transdermal TDLN-targeted delivery of R.E would enhance antitumor immunity and improve therapeutic outcomes compared to conventional approaches such as systemic and intratumoral drug deliveries (Scheme 1). The MN containing R.E (R.E@MN), consisting of an amphiphilic triblock copolymer, dissolves upon application to the tumor, releasing R.E-loaded nanomicelles (NMCs) (R.E@NMCs).^14^ R.E@NMCs encapsulate poorly water-soluble R.E within their core, enabling drug delivery to the TME and TDLN. Within the TDLN and TME, R.E@NMCs act as both an anticancer agent and an immune modulator, transforming the immunosuppressive TME into an immunostimulatory environment. This effect was characterized by a reduction in T_reg_ populations, polarization of tumor-associated macrophages (TAM) toward the pro-inflammatory M1 phenotype, and enhanced infiltration of effector T cells into the tumor, resulting in prolonged survival of the tumor-bearing mice.

**Scheme 1:**
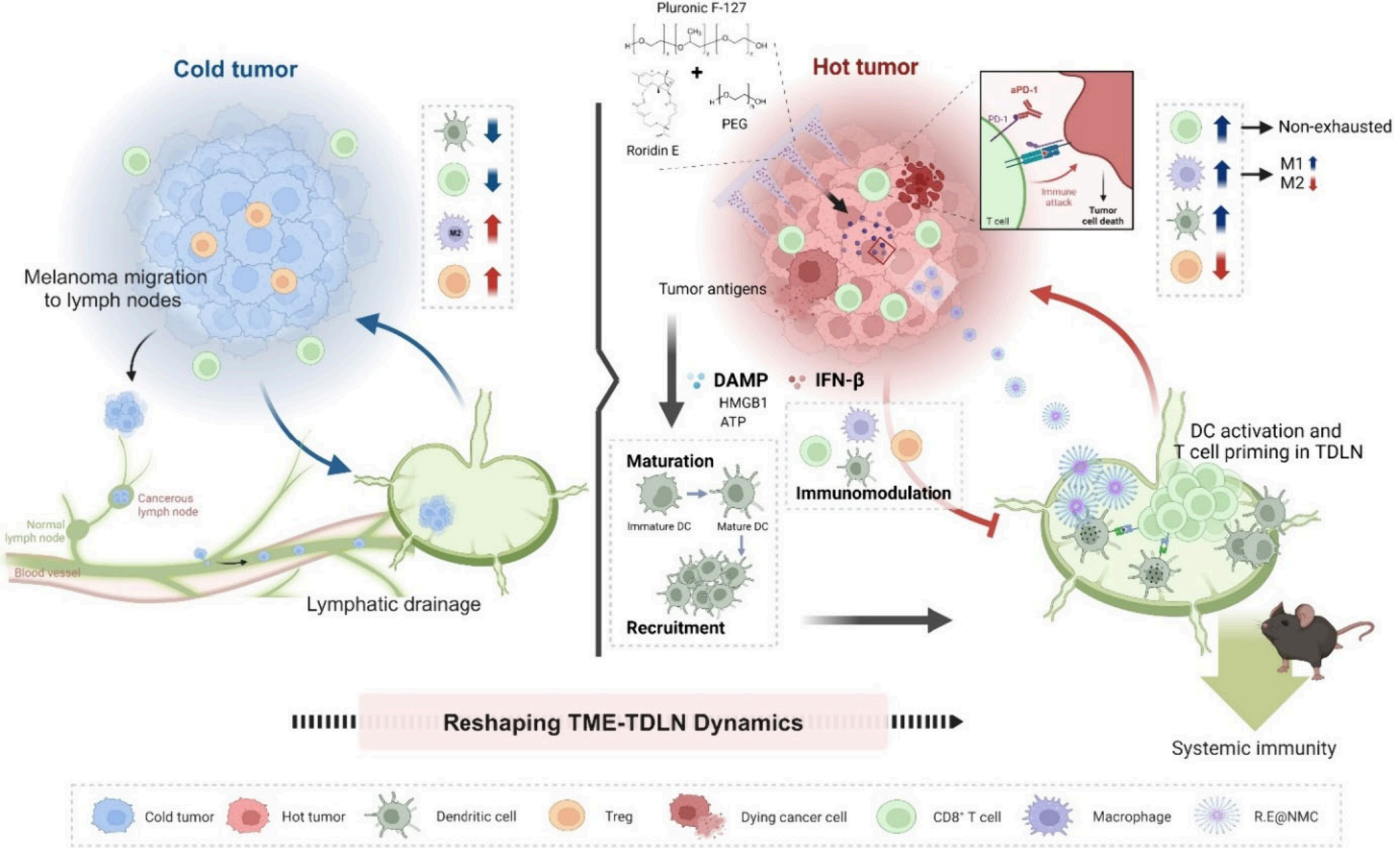
Schematic Illustration of Immune Modulation in Cancer Immunotherapy through Reshaping TME–TDLN Dynamics^a^

a The strategy leverages MN-mediated localized delivery of R.E@NMC to shift the TME from an immune-suppressive “cold” tumor state to an immune-active “hot” tumor state, effectively suppressing metastatic melanoma. R.E@MN treatment induces the release of tumor antigens, DAMPs such as HMGB1 and ATP, and IFN-β, which drive robust immune modulation. This promotes DC maturation, migration to TDLN, and efficient tumor antigen presentation, facilitating T cell priming in the TDLN. Consequently, R.E@MN treatment increases CD8^+^ T cell infiltration while reducing T_regs_ and M2 macrophages in the TME. When combined with aPD-1, this approach synergistically reinvigorates exhausted T cells, enhances cytotoxic T cell activity, and establishes systemic antitumor immunity.

The R.E-induced IFN-β production may cause potential contradictory effects in the TME. While IFN-β enhance cross-priming of dendritic cells and support survival of newly activated cytotoxic T lymphocytes (CTLs),^15^ it can also up-regulate PD-L1 on tumor cells through both IRF9-dependent and independent pathways.^16^ Thus, we hypothesized that R.E-induced IFN-β would stimulate antitumor immunity and transform a cold TME into a hot one, whereas the concurrent increase in PD-L1 could impose an adaptive inhibition on the function of CTLs. Combining R.E with an anti-PD-1 antibody (aPD-1) is expected to reduce the inhibition, reinvigorate exhausted CD8^+^ T cells, and enhance the therapeutic efficacy. The combination of the MN-guided localized delivery of R.E to the TME and TDLN with aPD-1 achieved synergistic effects, significantly improving tumor control, reducing metastasis, and overcoming resistance to ICIs, further supporting the potential of this approach in modulating the anticancer immunity in the TME–TDLN axis, thereby enhancing checkpoint blockade efficacy and overcome resistance. This strategy may offer significant translational potential by providing new therapeutic opportunities for patients who exhibit resistance to current ICI therapies.

## Results

### Isolation of Roridin E (R.E)

The methanol (MeOH) extract of *P. cornu-damae* cultures (Figure S1A,B, Supporting Information) was subjected to solvent partitioning using four organic solvents (hexane, CH_2_Cl_2_, EtOAc, and *n*-BuOH), yielding four main fractions. For the identification of R.E, each fraction was analyzed using LC–MS (Agilent 6545 Q-TOF LC/MS) and an in-house UV spectral library. The EtOAc-soluble fraction displayed a peak with the molecular formula C_29_H_38_O_8_ for R.E at *m*/*z* 537.2472 [M + Na]^+^ (calculated for C_29_H_38_O_8_Na, 537.2464). LC/MS-guided isolation led to the purification of R.E from the EtOAc fraction using semipreparative high-performance liquid chromatography (HPLC). The structure of R.E was confirmed through nuclear magnetic resonance (NMR) spectral comparison and LC/MS analysis (Figures S2 and S3A,B, Supporting Information).

### Anticancer and Immune-Modulating Properties of R.E

R.E has been previously reported to exhibit cytotoxic effects against human breast cancer cells.^17^ However, its broader biological effects have remained unexplored. We observed the dose-dependent cytotoxicity of R.E in B16F10 melanoma cells, but minimal effects on bone marrow-derived dendritic cells (BMDCs) and macrophages (BMDMs) (Figure 1A and Figure S4A,B, Supporting Information). To investigate the type of cell death, we first assessed R.E-induced immunogenic cell death (ICD) by measuring ATP and HMGB1 release, key damage-associated molecular patterns (DAMPs) that enhance immune activation. Remarkably, R.E induced higher levels of ATP and HMGB1 release than doxorubicin, a well-established ICD inducer (Figure 1B). Conditioned medium from R.E-treated B16F10 cells significantly increased the expression of costimulatory markers CD80 and CD86 on BMDCs, indicating its potential to activate DCs (Figure S5, Supporting Information). Based on the results, we hypothesized that R.E might have further immune modulatory properties and initially examined the R.E-induced transcriptional changes by performing RNA sequencing-based transcriptomic profiling on murine melanoma cells (B16F10) and murine embryonic fibroblast cells (NIH3T3) treated with R.E. We observed a substantial elevation in Ifnb1 gene expression, particularly in cancer cells (Supporting Information, RNA-seq data). Furthermore, R.E selectively induced IFN-β secretion in cancer cells (B16F10 and CT26 colorectal carcinoma) in a dose-dependent manner, while normal cells (NIH3T3 and HaCaT keratinocytes) showed no significant response, suggesting its tumor-specific immune modulatory properties (Figure 1C).

**Figure 1:**
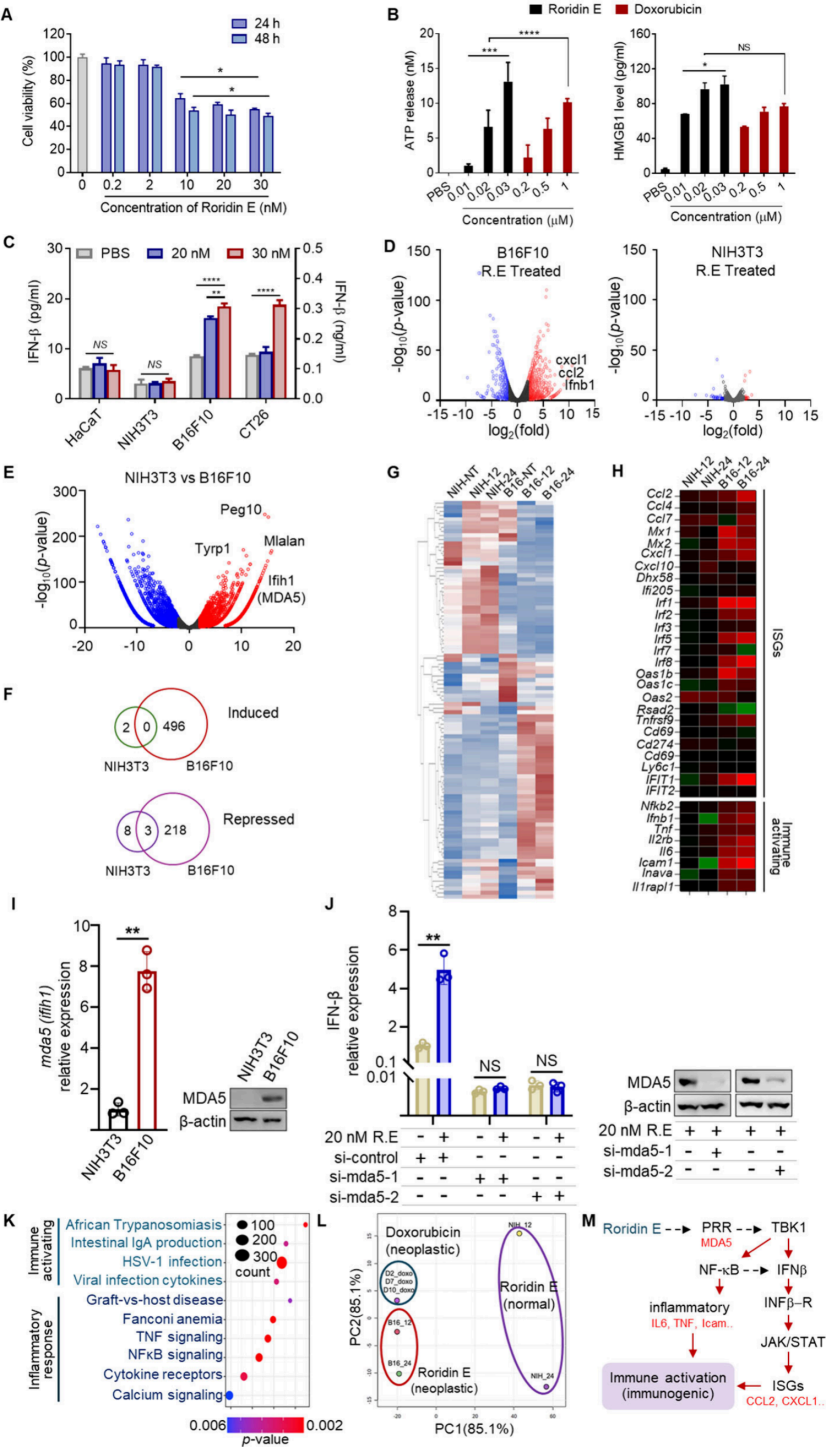
Immunogenic and transcriptomic alterations induced by R.E in B16F10 melanoma cells. (A) Dose-dependent cytotoxicity assay demonstrating the suppression of B16F10 melanoma cell growth by R.E at 24 and 48 h. (B) Release of ATP and HMGB1 in B16F10 cells treated with R.E or doxorubicin. (C) IFN-β release profiles across multiple cells following R.E treatment, showing significant IFN-β secretion in cancer cells (B16F10 and CT26) compared to normal cells (NIH3T3 and HaCaT). IFN-β levels measured in B16F10, NIH3T3, HaCaT cells (left axis, pg/mL) and CT26 cells (right axis, ng/mL) after 24 h of R.E treatment in a dose-dependent manner. (D) (left) Volcano plot displaying significant alterations in gene expression in B16F10 melanoma cells treated with R.E, highlighting extensive transcriptional changes. (right) Volcano plot for NIH/3T3 normal fibroblast cells treated with R.E, showing minimal transcriptional alterations. (E) Volcano plot comparing the induction of MDA5 in B16F10 melanoma cells and NIH3T3 normal cells, showing significant upregulation of MDA5 in melanoma cells. (F) Summary of the number of genes induced or repressed upon R.E treatment, demonstrating high selectivity toward B16F10 melanoma cells. (G) Hierarchical clustering analysis of untreated and R.E-treated NIH3T3 and B16F10 melanoma cells at 12 and 24 h post-treatment, showing distinct clustering patterns indicative of selective transcriptional responses. (H) Heatmap showing the induction of ISGs and proinflammatory genes in B16F10 melanoma cells treated with R.E, suggesting that R.E induces ISGs and immune activation-associated genes. (I) Quantitative analysis of MDA5 transcript (left) and protein (right) levels in NIH3T3 and B16F10 cells, showing MDA5 upregulation only in B16F10. (J) Analysis of IFN-β induction by R.E treatment upon MDA5 silencing for 18 h using siRNAs, demonstrating that IFN- β activation by R.E requires MDA5 (right). The silencing effect of the siRNAs was confirmed by measuring MDA5 protein expression upon siRNA treatment (left). (K) KEGG pathway analysis of B16F10 melanoma cells treated with R.E for 12 h, identifying significantly altered pathways related to microbial infection and immune activation. (L) Principal component analysis (PCA) of transcriptomic profiles in B16F10 melanoma cells treated with R.E compared to doxorubicin and normal cells, indicating that R.E induces a response similar to but distinct from doxorubicin. (M) Schematic summary of the proposed mechanism by which R.E potentially can act through MDA5 activation, leading to IFN-β induction and inflammatory responses. The induced IFN-β subsequently activates ISGs via the JAK/STAT pathway, and both ISGs and proinflammatory cytokines contribute to converting melanoma cells into immunogenic cells. Pairwise comparisons were assessed by Student’s *t* tests. Statistical significance was assessed using one-way ANOVA for multiple groups. All data are presented as mean ± SD *P < 0.05; **P < 0.01; ****P < 0.0001, NS = Not significant.

### Mechanistic Insights on Immune Modulation of R.E via Transcriptional Analysis

Transcriptomic profiling revealed significant transcriptional changes in R.E-treated B16F10 compared to NIH3T3 (Figure 1D,E). R.E altered the expression of 717 genes in melanoma cells (496 upregulated and 221 downregulated; *p* < 0.05, |log_2_(fold change)| > 2.25), while only 13 genes were affected in normal fibroblasts. Remarkably, no genes were commonly induced in both cell lines (Figure 1F), suggesting high selectivity of R.E. Clustering analysis of the RNA-seq results further confirmed this selectivity, with distinct patterns of transcript alterations for each cell line at 12 and 24 h time points (Figure 1G). R.E specifically induced 13 genes linked to immune responses against viral infections in melanoma cells (Figure 1H). These included (i) interferon-stimulated genes (ISGs) like CCL2, MX1, MX2, CXCL1, IRF1, IRF2, IRF5, IRF8, OAS1b, and IFIT1; (ii) chemokines such as CCL2, CCL4, and CXCL1 associated with leukocyte activation; and (iii) proinflammatory genes, including NFKB2, TNF, IL2RB, ICAM1, and IL6.

Type I IFNs are generally induced via two primary pathways: (1) the mitochondrial antiviral-signaling protein (MAVS) pathway, where cytosolic nucleic acid sensors such as RIG-I and MDA5 initiate signaling, or (2) the toll-like receptor (TLR) pathway, with receptors like TLR3 in the endosome. These pathways stimulate IFN-α/β production, subsequently activating interferon-stimulated genes (ISGs) through JAK/STAT signaling. We hypothesize that the selectivity of R.E for melanoma cells is due to the altered IFN pathway readiness in these cells. Baseline comparisons between NIH3T3 and B16F10 cells revealed more than 12-fold upregulation of MDA5 (encoded by *ifih1*) in melanoma cells (Figure 1E). This upregulation of MDA5 was confirmed by quantifying both the transcript and protein levels of MDA5 (Figure 1I). To further validate the mechanism of R.E through MDA5, we performed mRNA interference using siRNAs. Indeed, upon silencing MDA5 expression, the IFN- β induction by R.E was substantially reduced (Figure 1J). Pathway analysis of R.E-treated melanoma cells showed significant enrichment in pathways associated with microbial infection and immune activation (Figure 1K). These changes were observed only in melanoma cells and not in normal cells. Interestingly, this pattern of transcriptional change resembles that induced by doxorubicin, which triggers a signal relay to induce IFN-α by binding to TLR3, leading to IFN-α/β production and CXCL10 release.^18^ However, as shown in Figure 1L, while R.E-treated cancer cells had a transcriptional profile closer to that of doxorubicin-treated cells than to that of R.E-treated normal cells, the profile of R.E was distinct from that of doxorubicin. This suggests that R.E may induce IFN production through a receptor other than TLR3, potentially via MDA5, which is highly expressed in melanoma cells. In summary, our transcriptomic analysis suggests that R.E induces IFN-β production, which converts melanoma cells into an immunogenic state that releases proinflammatory cytokines, including CCL2 and CXCL1, potentially enhancing the recruitment of cytotoxic T cells (Figure 1M). In untreated conditions, melanoma cells produce minimal type I IFNs, suppressing IFN-related signaling and impairing cytotoxic T cell recruitment. However, R.E treatment reprograms melanoma cells to produce IFN, potentially through MAVS signaling associated with MDA5. This response is reminiscent of the immune activation seen with anthracycline drugs, which stimulate innate and T-cell-mediated anticancer responses.

The tumor-specific induction of IFN-β observed in Figure 1C further substantiates the transcriptome observation that melanoma cells exhibit increased readiness for IFN pathway activation, potentially due to the overexpression of receptors like MDA5. As shown in Figure 1D–H, R.E treatment enhanced pathways associated with immune activation, including upregulation of ISGs such as IRF1, MX1, and CXCL1, as well as pro-inflammatory cytokines including TNF and IL6 in melanoma cells. This specificity is further supported by the absence of shared gene induction between B16F10 and NIH3T3 cells, as indicated by the RNA-seq analysis (Supporting Information, RNA-seq data).

### Microneedle-Directed Targeting of the TME–TDLN Axis

To address the bidirectional communication within the TDLN–TME axis and minimize potential off-target toxicity and immune-related adverse effects, we employed an MN-based delivery system for localized administration. The MN based on an amphiphilic triblock copolymer was designed to dissolve upon dermal application and generate self-assembled NMCs suitable for migrating to TDLN 18. The MN, composed of poly­(ethylene oxide)-*b*-polypropylene oxide-*b*-poly­(ethylene oxide) (Pluronic F127) and polyethylene glycol (PEG), were prepared as illustrated in Figure 2A. PEG was employed to improve the mechanical properties of dissolving MN and enhance the micelle-forming capacity of F127 in intradermal fluid, as water molecules reduce their tendency to engage in hydrogen bonding with F127 in the presence of PEG,^19^ promoting micelle formation. To formulate poorly water-soluble R.E in the dissolving MN, R.E and F127 were dissolved in methanol. The solvent was then removed in a rotary evaporator to form a thin film. The final mixture for MN prepared by rehydrating the film in an aqueous solution containing PEG was applied to a poly­(dimethylsiloxane) (PDMS) mold and dried under reduced pressure to generate the microneedle structure. The rehydration process leads to the formation of block copolymer micelles, facilitating the dissolution of hydrophobic R.E in an aqueous milieu by localizing it to the hydrophobic core of the micelles, consisting of the poly­(propylene oxide) segment of F127. The backing layer supporting the microneedle shaft was made from poly­(vinyl alcohol) and polyvinyl pyrrolidone (PVA/PVP), which improved the blend miscibility and the flexibility of the layer owing to the formation of interchain hydrogen bonding.^20^ The MN array had 225 pyramid-shaped needles with 525 μm in a 0.8 × 0.8 cm patch. Figure 2B shows the morphology of the MN observed under stereomicroscope (upper) and scanning electron microscope (SEM, lower right) images. The confocal microscope image demonstrated that a fluorescent probe, Alexa Fluor 488, was confined within the needle shaft as opposed to the backing layer (Figure 2B, lower left). When MNs containing a hydrophobic fluorescent probe, 1,1′-dioctadecyl-3,3,3′,3′-tetramethylindodicarbocyanine, 4-chlorobenzenesulfonate salt (DiD), were applied to the back skin a mouse, SEM observation demonstrated that the needles were completely dissolved after 1 h (Figure 2C). The distribution of the NMCs encapsulating DiD (DiD@NMC) in the epidermal and dermal areas demonstrated the migration of the NMCs through the skin layers (Figure 2D). The MN can be readily dissolved in an aqueous medium, resulting in the generation of spherical NMCs with an average size of 78 nm (Figure 2E). The drug loading of R.E@NMC generated from the dissolution of R.E@MN was 0.38 ± 0.01% with an encapsulation efficiency of 75.31 ± 1.40%, as determined by HPLC analysis. The diameters of the NMCs less than 100 nm were known to be suitable for successful transition to and retention in lymph nodes, facilitating efficient lymphatic migration and stimulating resident immune cells (Figure 2F).^21^ *In vivo* observations demonstrated that the DiD@NMC migrated efficiently to TDLN within one hour of administration without significant systemic circulation, as evidenced by minimal distribution in major organs (Figure 2G). Immunohistochemical analysis using the dissected lymph node exhibited the colocalization of DiD@NMC with CD205^+^DCs and CD169^+^subcapsular macrophages (Figure 2H). These results suggested that the dermal application of MN resulted in the generation of NMCs that could efficiently deliver a hydrophobic small molecular drug to APCs in the TDLN through lymphatic vessels. Furthermore, the MN-guided localized delivery of R.E can also reduce the induction of systemic pro-inflammatory cytokine (IL-6), compared to intravenous (IV) and intratumoral (IT) administrations (Figure 2I). The reduced induction of systemic pro-inflammatory cytokine (IL-6) and stable blood biochemical parameters (Figure S6A,B, Supporting Information) suggest the effective reduction of the potential toxicities caused by systemic exposures to trichothecene mycotoxins while maintaining anticancer as well as immune modulating activities.

**Figure 2:**
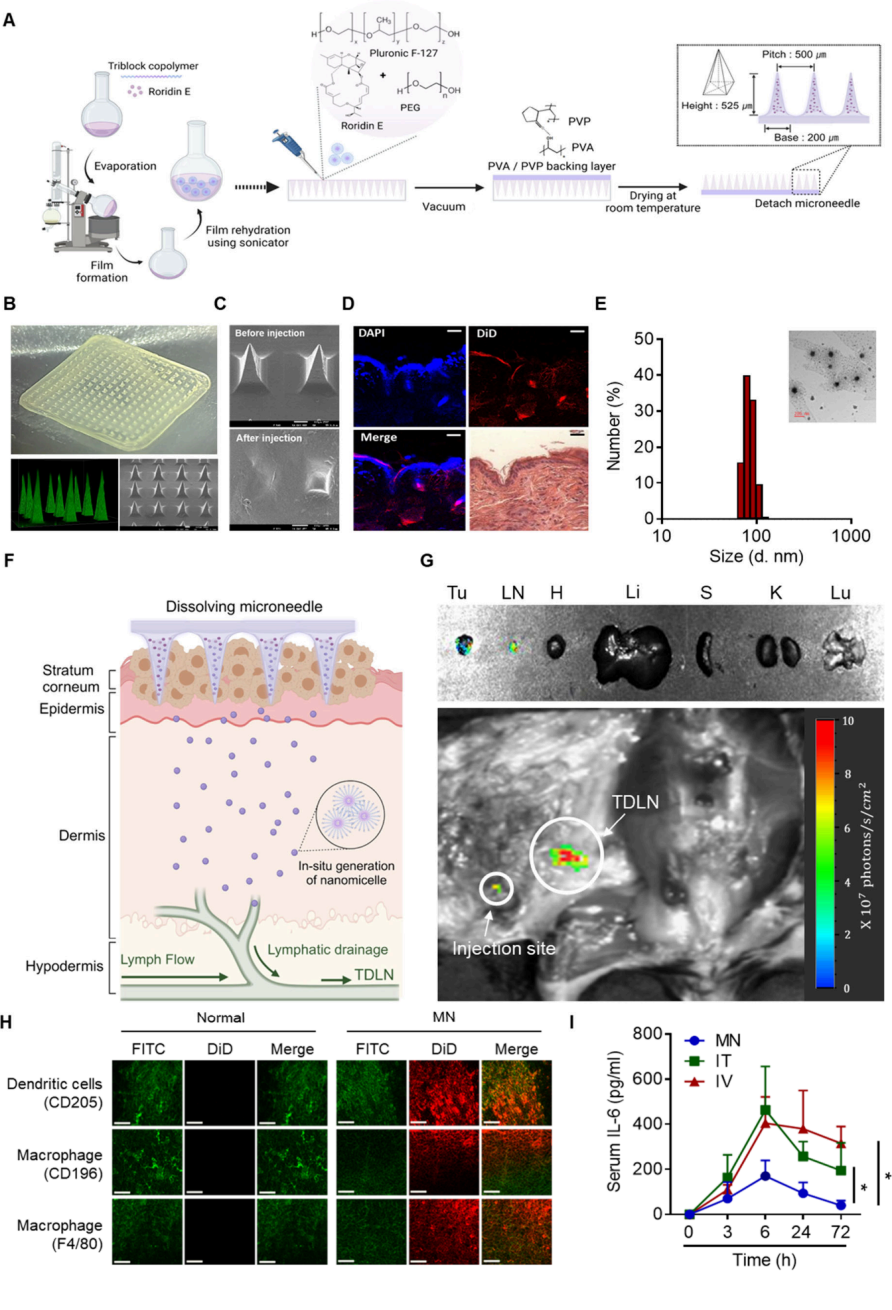
Engineering R.E@MN for targeted TDLN delivery and biological assessment. (A) Schematic illustration of the fabrication process of R.E@MN. (B) Morphology of the MN array visualized using stereomicroscope (upper panel), confocal microscopy (lower left), and scanning electron microscopy (SEM, lower right). (C) SEM images showing the intact structure of MN before application and its dissolution in the skin after 1 h of application on mouse back skin. (D) Intradermal delivery of DiD@NMC released from MN following the application to mouse skin. Fluorescence imaging shows that DiD signals (red) are distributed within the dermis layers, confirming successful penetration and localization. Cell nuclei are stained with DAPI (blue). Scale bars: 100 μm. (E) Size distribution and TEM image of NMCs formed upon MN dissolution in PBS. (F) Illustration depicting the *in situ* generation and release of drug-loaded NMC from MN in the skin, followed by lymphatic migration to TDLN. (G) Biodistribution of DiD@NMC released from MNs visualized by *in vivo* imaging. Distribution of DiD@NMC is observed in the TDLN and other organs (Tu: tumor; LN: lymph node; H: heart; Li: liver; S: spleen; K: kidney; Lu: lung). The injection site and TDLN were marked by white circles. (H) Immunohistochemical analysis of lymph nodes after the application of DiD@MN. Sections costained with DiD (red) and antibodies (green) against CD205 (dendritic cells, upper panel), CD169 (macrophages, middle panel), and F4/80 (macrophages, lower panel) confirm colocalization of NMCs with immune cells. Scale bars: 90 μm. (I) Serum IL-6 levels in tumor-bearing mice administered R.E via MN, IT, or IV routes. IL-6 levels were measured at 3, 6, 24, and 72 h postadministration. Statistical significance was assessed using one-way ANOVA for multiple groups. All data are presented as mean ± SD *P < 0.05.

### *In Vivo* Antitumor and Immune Modulation Effects of R.E in a Melanoma Model

Melanoma, a highly aggressive form of skin cancer, was chosen as the model to evaluate the antitumor efficacy of R.E *in vivo*. B16F10-bearing mice were treated with R.E via MN application (R.E@MN) and IT injections (R.E­(IT)) using hypodermic syringe at two-day intervals (Figure S7A, Supporting Information). Mice treated with high-dose R.E­(IT) and R.E@MN showed significant tumor inhibition and prolonged survival, with overall survival rates of 60% beyond 40 days (Figure S7B–D, Supporting Information). Flow cytometry revealed an increase in CD8^+^ T cell infiltration in both the tumor and TDLN following MN treatment (Figure S8, Supporting Information), suggesting that R.E@MN enhances tumor-specific immune responses. The intricate interactions among several TME components substantially influence the prognosis and response to ICI treatment.^6,22^ Factors that promote T cell infiltration, activation, and function generally enhance ICI efficacy, while immunosuppressive elements in the TME can limit ICI responses. In addition, the dynamic interplay between the TME and TDLN creates a unique immune-tolerant niche that often contributes to the systemic spread of cancer cells. We hypothesized that microneedle (MN)-guided intratumoral and lymphatic delivery of R.E could target both the TME and TDLN, enabling the simultaneous disruption of immunosuppressive pathways and activation of pro-inflammatory immune responses at both sites. To investigate the potential of R.E to modulate and restore antitumor immunity, we combined it with an anti-PD-1 antibody (aPD-1), as targeting the PD-1/PD-L1 axis is typically effective in the presence of functional T cells within the TME. We anticipated that this combination would reshape the immune landscape within the TME and TDLN, enhancing immune cell infiltration and activity while reducing the influence of immunosuppressive cells, thereby shifting the TME from an immunologically “cold” to a “hot” state. To investigate the effects of the combination of R.E with aPD-1 on the tumor growth, R.E@MN was administered to the tumor site, with aPD-1 given intraperitoneally as per the schedule in Figure 3A. The combination treatment of R.E@MN with aPD-1 led to significantly greater tumor inhibition compared to monotherapies with R.E@MN, R.E­(IT), or aPD-1 alone, as well as the PBS control group. Tumor weight and body weight were also measured to assess tumor progression and potential cytotoxicity (Figure 3B–E and Figure S9, Supporting Information). These results suggest that the combined R.E@MN and aPD-1 treatment may exert a synergistic effect in suppressing primary tumor growth.

**Figure 3:**
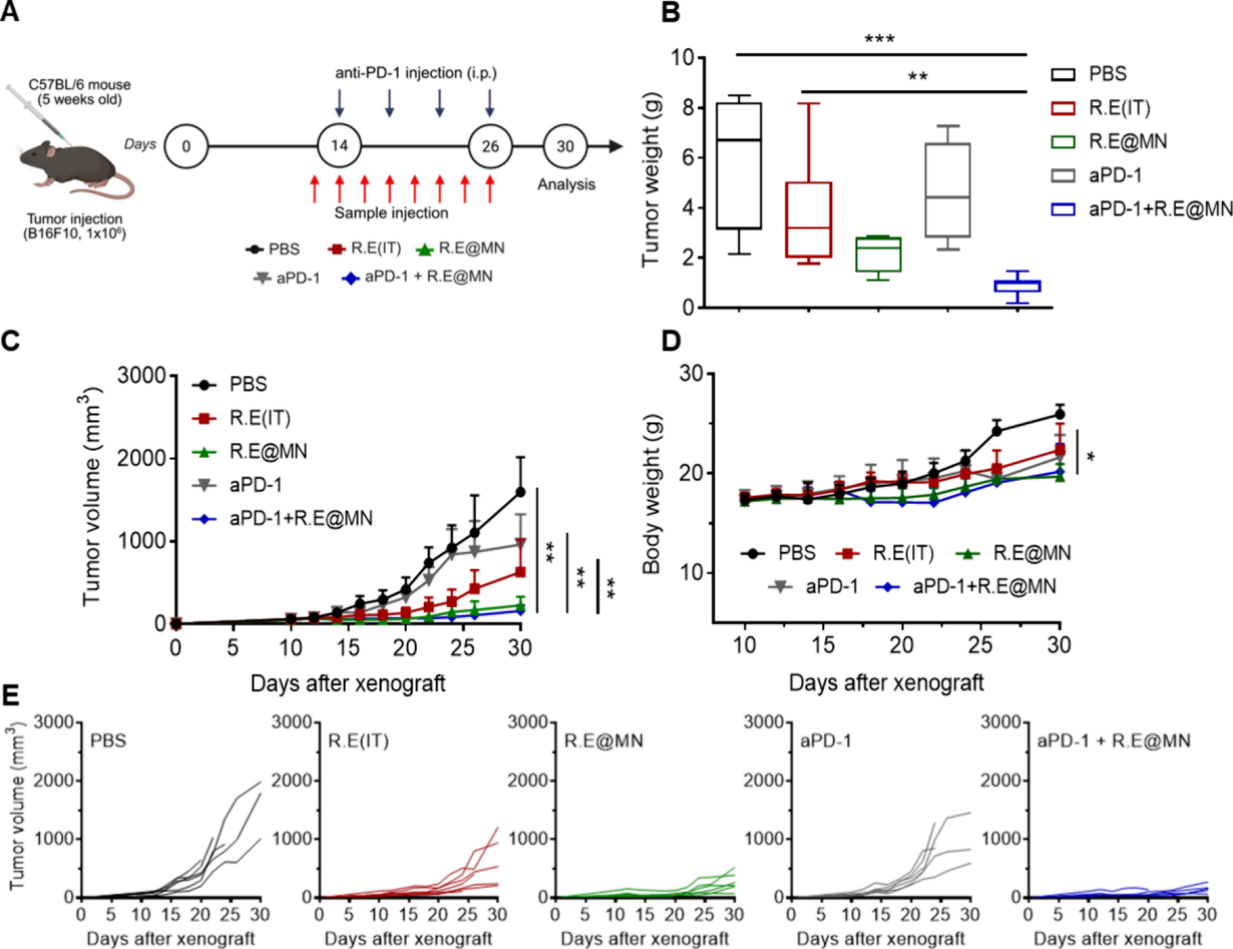
R.E and aPD-1 enhances therapeutic efficacy in a primary tumor model. (A) Treatment schedule outlining R.E administration via IT injection or MN application every 2 days, with intraperitoneal administration of aPD-1 initiated on day 14 post-tumor inoculation. (B) Tumor weight at the end point (day 30) across different treatment groups, demonstrating significant tumor reduction in the R.E@MN + aPD-1 group compared to other groups. (C) Average tumor growth curves showing the inhibition of tumor growth in mice treated with R.E, R.E@MN, aPD-1, and R.E@MN + aPD-1. (D) Body weight monitoring during the treatment period, showing no significant weight loss, suggesting low systemic toxicity of the combination therapy. (E) Individual tumor growth curves for each treatment group (*n* = 6 per group). Statistical significance was assessed using one-way ANOVA for multiple groups. All data are presented as mean ± SD *P < 0.05; **P < 0.01; ***P < 0.001.

Given the potential for cancer cells to migrate into lymphatics and communicate with immune cells in TDLN, we examined immune cell populations within both the tumor and TDLN to evaluate the effects of each treatment group. Although all the treatment groups, including R.E­(IT), R.E@MN and aPD-1, showed a significant increase in CD8^+^ T cell to both the tumor and TDLN, the combination of R.E@MN with aPD-1 resulted in a remarkably higher infiltration of CD8**^+^** T cells in both sites, compared to the monotherapies (Figure 4A,B). In addition, the remarkable reduction of regulatory CD4**^+^** CD25**^+^** FoxP3**^+^** T cells (T_regs_) was also observed in all treatment groups (Figure 4C,D). While the combination of R.E@MN with aPD-1 showed significant synergistic effects in promoting CD8**^+^** T cell recruitment and T_reg_ depletion, it should be noted that both R.E and R.E@MN also exhibited immune-modulating properties. This can be attributed to the unique mechanism of action of R.E involving IFN-β induction in cancer cells (Figure 1C–J). The transient increase of IFN-β in TME could lead to the suppression of activation and proliferation of T_regs_ either by direct pro-apoptotic and antiproliferative effects on T_regs_ ^23^ or by reducing interleukin-2 (IL-2) production which is critical for the proliferation, survival, and function of T_regs_.^24^ The effective suppression of T_regs_ in TME can further influence CD8**^+^** T effector cells by enhancing their function and proliferation and reversing exhausted phenotypes.^25^ Therefore, we evaluated the effect of R.E treatments on T cell exhaustion by profiling exhausted CD8**^+^** PD-1**^+^** T cells in the tumor and TDLN 1 week after treatment as scheduled in Figure 4E. The decrease in the exhausted T cells became more significant as time goes on in the tumor and TDLN (Figure 4F and Figure S10, Supporting Information). Both the R.E@MN and the combination treatment groups showed a significant reduction in the number of exhausted T cells a week after the administration compared to the control group (Figure 4G), suggesting a shift toward a more pro-inflammatory immune environment allowing the enhanced infiltration of functionally active CD8**^+^** T cells.

**Figure 4:**
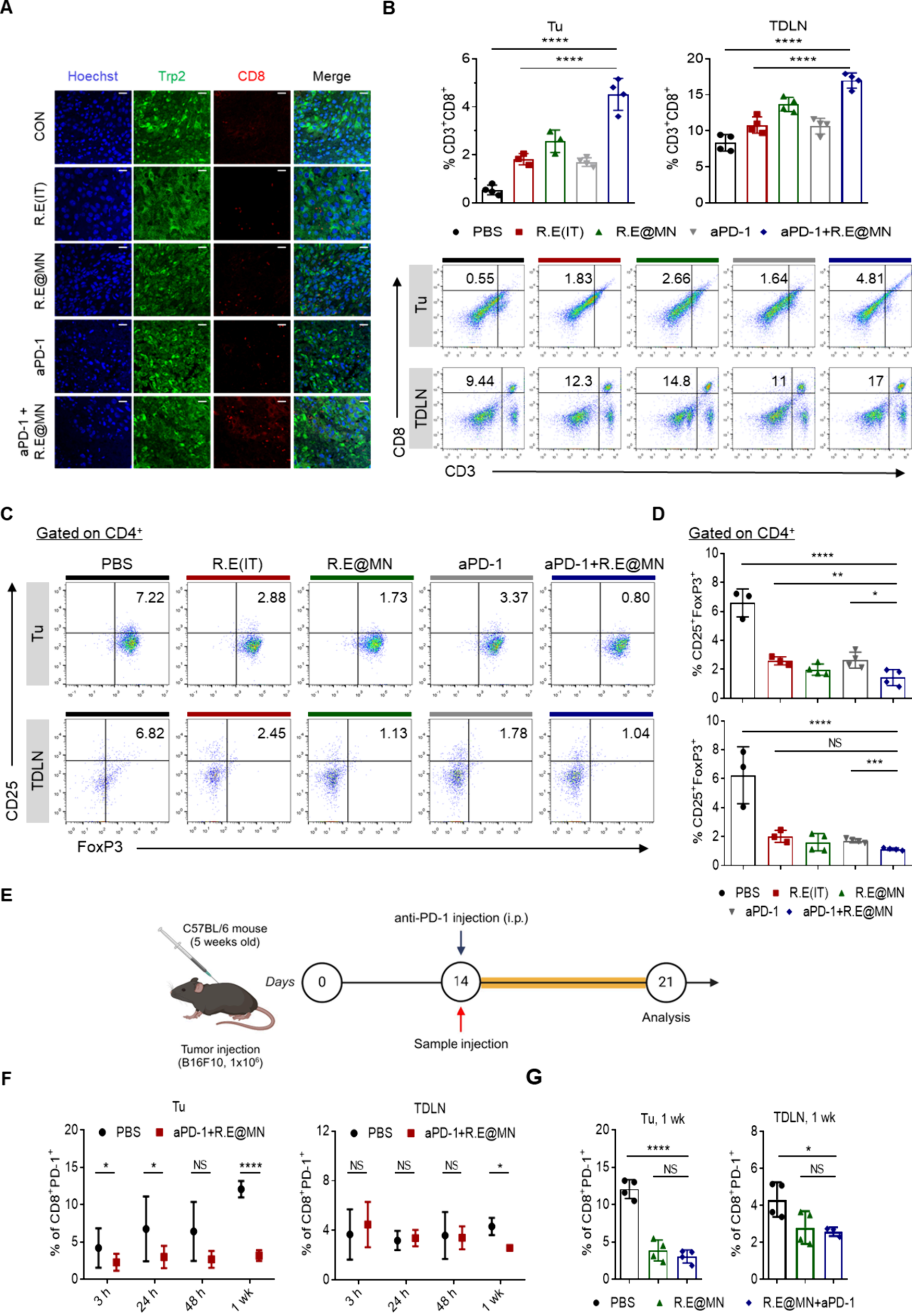
R.E@MN enhances antitumor immunity by modulating TDLN–tumor immune crosstalk and exhibits synergistic effects when combined with aPD-1 in a B16F10 melanoma model. (A) Representative confocal images illustrating CD3^+^CD8^+^ T cell infiltration within tumor tissues in the treatment groups. Scale bars, 20 μm. (B), Quantitative and flow cytometry analysis of CD3^+^CD8^+^ T cell infiltration in tumors (Tu) and TDLN, demonstrating increased infiltration in R.E@MN and R.E@MN + aPD-1 groups compared to controls. (C) Flow cytometry plots showing CD4^+^CD25^+^FoxP3^+^ T_reg_ populations in Tu and TDLN in the treatment groups. (D) Quantitative analysis of T_reg_ populations (CD25^+^FoxP3^+^ gated on CD4^+^) in Tu and TDLN, demonstrating significant reduction in R.E@MN and aPD-1 combination groups. (E) Experimental timeline for assessing T cell persistence and exhaustion dynamics over 1 week post-treatment. (F) Proportions of CD8^+^PD-1^+^ T cells in Tu and TDLN evaluated at various time points (3, 24, 48 h, and 1 week), showing reductions in T cell exhaustion in the R.E@MN and aPD-1 combination groups. (G) Comparison of CD8^+^PD-1^+^ T cell populations in Tu and TDLN 1-week post-treatment, suggesting reduced exhaustion levels in the R.E@MN and aPD-1 combination groups. (*n* = 3–4 per group). Statistical significance was assessed using Student’s *t*-tests for pairwise comparisons and one-way ANOVA for multiple groups. All data are presented as mean ± SD *P < 0.05; **P < 0.01; ***P < 0.001; ****P < 0.0001, NS = Not significant.

The transcriptomic and *in vivo* studies in melanoma-bearing mice demonstrated that R.E induces a robust IFN-β response in tumor. The increased population of TRP2^+^IFN-β^+^ melanoma cells from tumor tissue exhibited that R.E can induce IFN-β production in tumor cells *in vivo* (Figure S11, Supporting Information). This response was more significant in groups treated with R.E@MN and in combination with aPD-1 compared to the R.E and aPD-1 monotherapy groups (Figure 5A). The production of IFN-β by cancer cells within the TME is noteworthy, as type I interferons like IFN-β are typically produced by immune cells, including dendritic cells (DCs) and macrophages, in response to external stimuli such as viral infections or DAMPs.^26^ IFN-β plays a crucial role in modulating the immune landscape of the TME by promoting the polarization of TAMs from an immunosuppressive M2 phenotype to a pro-inflammatory M1 phenotype (Figure 5B,C). These results are consistent with prior studies showing that type I IFNs, including IFN-β, can inhibit M2 macrophage activity and promote M1 polarization, which is crucial for creating an immune-activated TME.^27^ The inhibition of IFN-β signaling by antibody targeting interferon α and β subunit 1 receptor (anti-IFNAR) resulted in the reduction in the tumor growth suppression (Figure S12A,B, Supporting Information), indicating the significant contribution of IFN-β in the tumor growth inhibition by R.E. The reduction of CD45^+^IFN-β^+^ cells after the administration of anti-IFNAR suggests that the induction of IFN-β in the CD45^+^ immune cells would be due to paracrine effects rather than direct stimulation of the cells by R.E suppression (Figure S12C, Supporting Information). In addition, IFN-β induced by R.E@MN enhances the recruitment of activated DCs within the tumor, characterized by the upregulation of costimulatory molecules essential for effective priming of T cells in the TME (Figure 5D and Figure S13, Supporting Information). The activation of CD11c^+^ cells was observed as early as 3 h following treatment. This activation was sustained for up to 1 week within the tumor and extended to the TDLN (Figure 5E). In addition to the action of IFN-β, the R.E-induced immunogenic death of cancer cells releasing ATP and HMGB1 (Figure 1B) can also contribute to the recruitment and activation of DCs in the TME and promote the uptake of dying tumor cells and presentation of tumor antigens by DCs.^28^ This enhanced antigen presentation and T cell activation in the TME^29^ and TDLN^30^ would further evoke the systemic anticancer immunity. To assess the effects of R.E treatment on systemic immunity, we measured IFN-γ-secreting CD8^+^ T cells in the spleen to evaluate the broader immune response. Flow cytometry analysis showed a significant increase in IFN-γ-expressing CD3^+^CD8^+^ T cells responsive to tumor antigen gp100 in the R.E@MN and R.E@MN/aPD-1 combination groups compared to the PBS control (Figure 5F,G and Figure S14, Supporting Information). Moreover, the combination of R.E@MN with aPD-1 significantly reduced both lung and lymph node metastasis, as observed in histological analyses (Figure 5H and Figure S15, Supporting Information). This reduction in metastatic spread suggests the synergistic effect of R.E with aPD-1, where R.E-mediated immune modulation and PD-1 blockade collaborate to suppress tumor cell dissemination and promote immune surveillance in distant tissues (Figure 5H). These findings demonstrated that the R.E, particularly when administered through the MN-based system and in combination with aPD-1, can effectively reprogram the TME. R.E promotes macrophage polarization to the M1 phenotype, enhances DC activation, and induces the recruitment and activation of functional effector T cells. These changes reshape the anticancer immunity by converting the TME and TDLN from a suppressive to a more immunologically active state, ultimately leading to better tumor control, reduced metastasis, and improved survival outcomes in the treated mice (Figure 5I,J).

**Figure 5:**
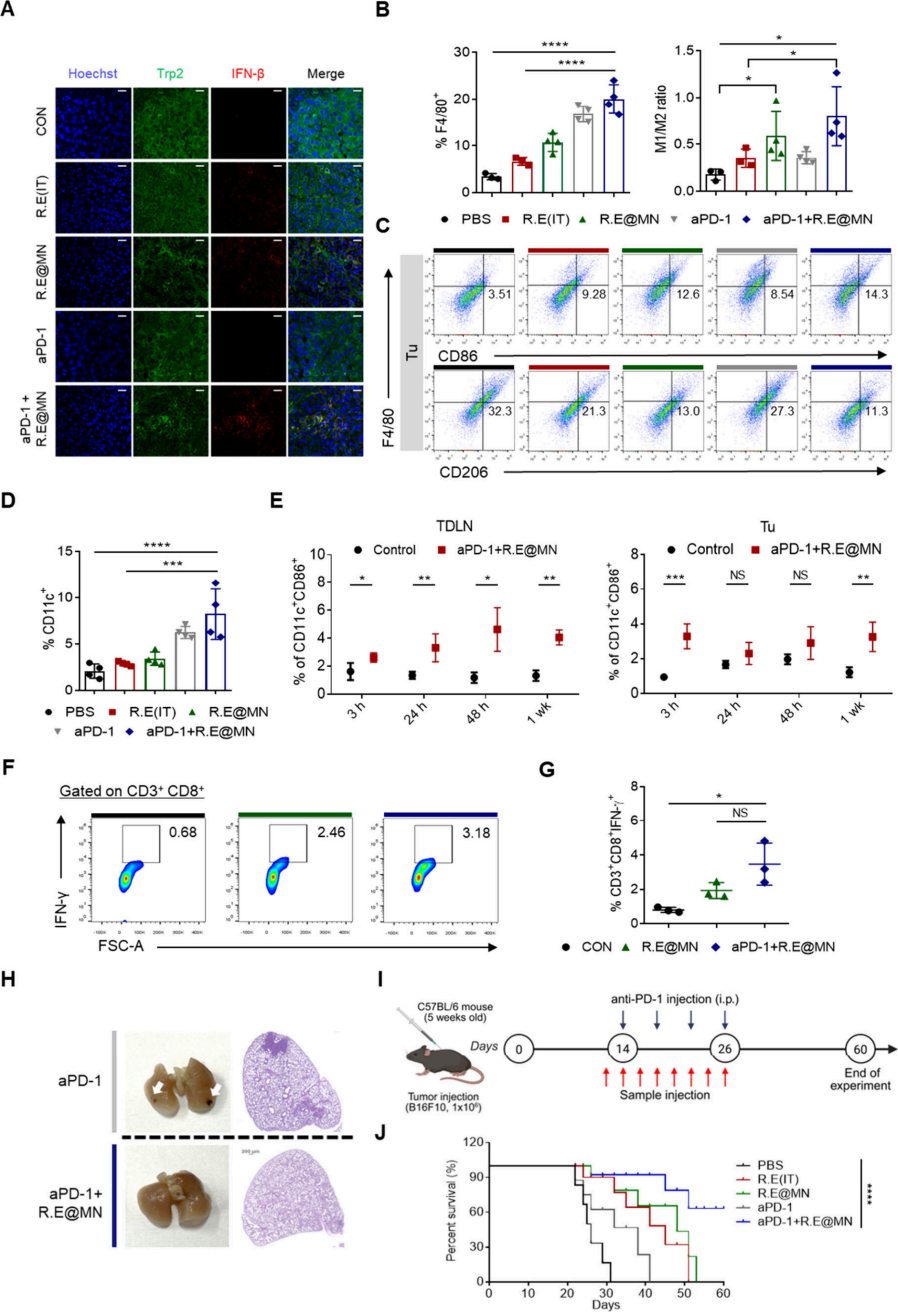
IFN-β-mediated and immune modulation and recruitment of immune cells in the tumor microenvironment following R.E@MN and aPD-1 treatment. (A) Representative confocal images showing IFN-β accumulation within tumors. Tumor sections were stained with Hoechst 33258 (nucleic acid, blue), anti-TRP2/DCT (melanoma marker, green), and anti-IFN-β (red). Scale bars, 20 μm. (B) Recruitment of macrophages (left panel) and M1/M2 macrophage ratio in tumor, showing polarization of TAM with F4/80^+^CD86^+^ (M1) and F4/80^+^CD206^+^ (M2) markers (right panel). (C) Flow cytometry analysis of TAM polarization after treatments. (D) Flow cytometry analysis of CD11c^+^ dendritic cells (DCs) in tumor tissues, indicating increased DC recruitment following the treatment with R.E@MN and aPD-1 + R.E@MN combination. (E) Changes of CD86^+^CD11c^+^ DC populations in TDLN and tumors, indicating temporal activation of DCs in response to aPD-1 + R.E@MN combination. (*n* = 3–4 per group). (F) Representative flow cytometry plots showing IFN-γ^+^ CD8^+^ T cell populations in splenocytes stimulated with gp100 antigen. (G) Fraction of antigen-specific IFN-γ^+^CD8^+^ T cells in spleen, confirming enhanced antigen-specific T cell activation in the R.E@MN + aPD-1 group (*n* = 3 per group). (H) Assessment of lung metastasis: macroscopic lung images and H&E-stained sections showing metastatic foci (white arrows). Scale bars, 200 μm. (I) Schematic representation of the treatment schedule. (J) Survival curves for tumor-bearing mice across treatment groups, monitored over 60 days (*n* = 12–15 per group). Statistical significance was assessed using Student’s *t* tests for pairwise comparisons and one-way ANOVA for multiple groups. All data are presented as mean ± SD * *P* < 0.05; **P < 0.01; *** *P* < 0.001; ****P < 0.0001, NS = Not significant.

## Discussion

In this study, we report that an MN-based transdermal lymphatic delivery system for a potent immune modulator, a natural macrocyclic trichothecene mycotoxin (R.E), achieves robust antitumor efficacy by reprogramming the tumor-lymph node immune axis. R.E exhibits dual functionality by inducing ICD and stimulating the secretion of IFN-β directly from cancer cells. To our knowledge, this is the first demonstration that a trichothecene mycotoxin can activate tumor-intrinsic IFN-β production, a property that satisfies the current repertoire of ICD inducers.

*In vitro* studies found that R.E treatment not only induces dose-dependent cytotoxicity in B16F10 melanoma cells but also stimulates the release of DAMPs, ATP and HMGB1. It should be noted that R.E uniquely triggers IFN-β secretion in cancer cells without affecting normal cells. Transcriptomic analysis reveals that R.E selectively reprograms the transcriptome of B16F10 melanoma cells with a marked upregulation of interferon-stimulated genes (ISGs) and chemokines (e.g., CXCL1, CCL2) associated with antiviral responses, which were not observed in normal fibroblasts. Notably, the RNA-seq results showed a significant increase in the expression of MDA5 (encoded by *ifih*1), suggesting that R.E may activate innate immune pathways-possibly through a RIG-I/MDA5–MAVS axis, which then activates IRF3 and NF-κB to promote IFN-β production. This “viral mimicry” response is similar to what was observed with anthracyclines^18,31^ and other DNA-damaging agents,^32^ where tumor cells take on an antiviral state that enhances antigen presentation and T cell recruitment. Some conventional chemotherapeutic agents, such as doxorubicin and oxaliplatin, have been reported to induce ICD by inducing the release of DAMPs and autocrine IFN-β signaling in tumor cells.^18,32^ Similarly, poly­(I:C) and RIG-I agonists directly engage cytosolic RNA sensors to trigger type I interferon production.^33^ While these agents effectively turn dying tumor cells into a source of immune stimulatory signals, R.E is distinct in that it comes from a natural product and appears to selectively target cancer cells for IFN-β induction. This selective activation is underscored by our observation that normal fibroblasts and keratinocytes are mostly unresponsive to R.E. Thus, R.E not only provides the desirable features of established ICD inducers but also may have improved specificity, potentially reducing off-target toxicities. It should be noted that when type-I IFN signaling was inhibited with anti-IFNAR1 mAb, we still observed the suppression of tumor growth (Figure S12B), demonstrating the similar pattern reported for doxorubicin-induced ICD.^34^ The results suggest that cytotoxicity and immunogenicity are complementary, rather than mutually exclusive. R.E may accomplish the immediate elimination of susceptible cancer cells, while the subsequent MDA5-dependent secretion of IFN-β facilitates dendritic-cell cross-priming and sustained CD8^+^ T-cell-mediated tumor control.

The NMC-generating MN-based delivery system plays a critical role in the therapeutic efficacy of R.E. The MN platform facilitates localized, transdermal delivery, ensuring that R.E-loaded nanomicelles (R.E@NMCs) effectively target the TME–TDLN axis. This dual targeting reduces systemic exposure and related toxicities, as demonstrated by decreased blood IL-6 levels compared with intravenous (IV) or intratumoral (IT) injections. Furthermore, the localized delivery enables a focused immune-modulatory environment that facilitates DC maturation, macrophage polarization toward the M1 phenotype, and enhanced recruitment of cytotoxic CD8^+^ T cells. The MN system therefore serves not only as a means of drug delivery but also as a strategic tool for reshaping the immune landscape of both the TME and TDLN.

*In vivo* experiments demonstrate that the combination of MN-mediated R.E delivery with anti-PD-1 therapy yields superior tumor suppression and survival benefits compared to monotherapies. The R.E-induced IFN-β response seems to contribute to enhanced T cell priming and activation.^15^ However, it may also upregulate immune checkpoints such as PD-L1 on tumor cells.^16^ This adaptive resistance mechanism suggests the rationale for combining R.E with ICIs. By concurrently reducing T cell exhaustion and enhancing antigen presentation, the combined therapy effectively transforms an immunologically “cold” tumor into a “hot” one, thereby overcoming one of the major hurdles in current cancer immunotherapy.

Our findings provide strong proof that MN-guided lymphatic delivery of R.E represents a promising strategy for cancer immunotherapy. By leveraging the dual functionality of R.E, this approach not only enhances local immune activation but also primes systemic antitumor responses. The selectivity of R.E for cancer cells, together with the targeted delivery provided by the MN system, could offer significant translational potential. Future studies should aim to elucidate the precise molecular mechanisms underlying R.E-induced IFN-β production and explore its applicability across different tumor types. Additionally, investigating the combination of R.E with other ICIs or immunomodulatory agents may further expand its clinical applicability.

In conclusion, this study demonstrates that reprogramming the tumor-lymph node immune axis via the localized delivery of a natural immune modulator can overcome important huddles in cancer immunotherapy. The ability of R.E to induce a robust IFN-β response from tumor cells highlights a new mechanism of action that, when combined with advanced drug delivery systems, may pave the way for more effective and less toxic immunotherapeutic regimens.

## Supplementary Material



## Notes

“Abbreviations”, “Materials and Methods” (experimental details for cell and animal procedures, transcriptomics workflow and analysis, microneedle fabrication/characterization, biodistribution, cytokine/toxicity assays, flow cytometry, and statistics) and additional figures: Photograph of *P. cornu-damae* and chemical structure of roridin E (Figure S1); HR-ESIMS spectrum of roridin E (Figure S2); ^1^H NMR and ^13^C NMR spectra of roridin E (Figure S3); Dose-dependent cytotoxicity of roridin E on BMDMs and BMDCs (Figure S4); BMDC maturation (CD80/CD86) after incubation with conditioned medium from roridin E-treated B16F10 cells (Figure S5); Blood biochemistry and hematology after treatment (Figure S6); Therapeutic efficacy of roridin E in melanoma (tumor growth, body weight, survival) (Figure S7); Flow-cytometric analysis of CD3^+^CD8^+^ T-cell infiltration in tumors and TDLNs (Figure S8); Representative excised tumors (Figure S9); Gating strategy for flow cytometry analysis of exhausted T cells (CD3^+^CD8^+^/PD-1^+^) (Figure S10); IFN-β quantification and confocal images of tumor sections after roridin E treatment (Figure S11); Anti-IFNAR1 blocking study of tumor growth and type I IFN signaling (Figure S12); DC maturation in the TME (Figure S13); Gating strategy for IFN-γ-producing CD8^+^ T cells in spleen (Figure S14); Representative lung and lymph node sections showing metastatic foci (Figure S15) (PDF)
