# Supplementary material for: Reshaping Tumor-Lymph Node Immune Axis via Targeted Lymphatic Delivery of Dual-Functional Immune Modulator for Enhanced Cancer Immunotherapy
Source: ACS Cent Sci. 2025 Sep 17;11(11):2074–86. doi: 10.1021/acscentsci.5c00509 (PMC12670315; doi:10.1021/acscentsci.5c00509)
Supplement: Supplementary file 1 [file oc5c00509_si_001.pdf]

Supporting Information

Reshaping Tumor-Lymph Node Immune Axis via Targeted Lymphatic Delivery of Dual-Functional Immune Modulator for Enhanced Cancer Immunotherapy

Su Yeon Lim[†], Pin Liu[†], Ju Hwa Shin[†], Bum Soo Lee[†], Sun Ju Kim[†], Dahwun Kim[†], Siyan Lyu[†], Byung Deok Kim[†], Chaeeun Park[†], Junku Jung[†], Jihyun Lee[†], Jinbeom Seo[†], Taegwan Yun[†], Hyo Jin Park[†], Min Sang Lee[†,#], Ki Hyun Kim[†,], Wonsik Lee[†,*] and Ji Hoon Jeong[†,ǁ,§,*]*

[†]School of Pharmacy, Sungkyunkwan University, Suwon 16419, Republic of Korea

[ǁ]Department of MetaBioHealth, Institute for Cross-disciplinary Studies, Sungkyunkwan University, Suwon 16419, Republic of Korea

[§]Biomedical Institute for Convergence at SKKU, Sungkyunkwan University, Suwon 16419, Republic of Korea

[#]Gyeonggido Business and Science Accelerator, Suwon-si, Gyeonggi-do 16229, Republic of Korea

[*]Correspondence should be addressed to khkim83@skku.edu (K. H. Kim); wonsik.lee@skku.edu (W. Lee); jhjeong@skku.edu (J. H. Jeong)

ABBREVIATIONS: aPD-1, anti-programmed cell death-1 antibody; APC, antigen-presenting cell; BMDM, bone-marrow-derived macrophage; BMDC, bone-marrow-derived dendritic cell; DAMP, damage-associated molecular pattern; ICD, immunogenic cell death; ICI, Immune checkpoint inhibitors; IFN-β, interferon-β; IFN-I, type-I interferon; IT, intratumoral; IV, intravenous; NMC, nano-micelle; MDA5, melanoma differentiation-associated protein 5; MN, microneedle; PEG, poly(ethylene glycol); R.E, roridin E; R.E@MN, roridin E-loaded microneedles; R.E@NMC, roridin E-loaded nano-micelle; TDLN, tumour-draining lymph node; TME, tumour microenvironment;

MATERIALS AND METHODS

Materials

Poly(vinyl alcohol) (PVA, Mw = 8,500), polyvinylpyrrolidone (PVP, Mw = 10,000), Pluronic F127 (F127), and neutral buffered formalin solution (10%) were purchased from Sigma-Aldrich (St. Louis, MI, USA). Polydimethylsiloxane (PDMS) MN molds were obtained from M Patch (Micropoint Technologies Pte Ltd, Singapore). Dulbecco's phosphate-buffered saline (PBS), Dulbecco's modified Eagle's medium (DMEM), and RPMI 1640 medium were purchased from Welgene (Gyeongsan, Korea), while trypsin-EDTA was acquired from Gibco (Waltham, MA, USA). Hoechst 33258 and DiD (1,1'-Dioctadecyl-3,3,3',3'-Tetramethylindodicarbocyanine, 4-Chlorobenzenesulfonate Salt) were obtained from Invitrogen (Carlsbad, CA, USA). The optimal cutting temperature (OCT) compounds were obtained from Sakura Finetek (Torrance, CA, USA). Tissue embedding molds were obtained from Polysciences (Warrington, PA, USA). Micro-slide glasses were purchased from Matsunami (Osaka, Japan). All chemicals and solvents used were of analytical grade and utilized without further purification.

Mice and Cells

Five-week-old female C57BL/6 mice were purchased from Orient Bio (Seongnam, Korea). The mice were housed under controlled environmental conditions, including a temperature of 22–25 °C, 55–60% relative humidity, and a 12-hour light/dark cycle. All animal experimental protocols were reviewed and approved by the Institutional Animal Care and Use Committee (IACUC) of Sungkyunkwan University School of Medicine and were conducted in accordance with institutional guidelines under the oversight of the SKKU School of Pharmacy. B16F10, NIH3T3, CT26, and HaCaT cell lines were obtained from the Korean Cell Line Bank (KCLB, Seoul, Korea) and cultured in high-glucose DMEM supplemented with 10% fetal bovine serum (FBS), 100 U mL^{-1} penicillin, and 100 µg mL^{-1} streptomycin in a humidified 5% CO_2 incubator at 37 °C. Bone marrow-derived dendritic cells (BMDCs) and macrophages (BMDMs) were isolated from the femurs of 5-week-old C57BL/6 mice. Red blood cells were lysed using RBC lysis buffer (eBioscience, Thermo Fisher Scientific, Waltham, MA, USA). BMDCs were cultured with 20 ng mL^{-1} GM-CSF, and BMDMs with 20 ng mL^{-1} M-CSF in RPMI media. Cells were collected on day 7 for further flow cytometric analysis, achieving >85% $CD11c^+$ or $F4/80^+$ as a phenotypic marker for non-adherent DCs and macrophages, respectively.

Transcriptome analysis

To find transcriptional changes in B16F10 melanoma cells and NIH3T3 fibroblasts upon treatment of R.E, we performed transcriptome profiling. First, both cell lines were treated with R.E or phosphate buffered saline (PBS) and incubated at 37 °C for 12 or 24 hours, then total RNA was isolated using RNeasy plus mini kit (Qiagen, Düsseldorf, Germany) (PMID:31866224). The extracted total RNA was sequenced with TruSeq stranded mRNA library kit using the NovaSeq platform at Macrogen (Seoul, South Korea). For data processing, the raw data were quality checked and trimmed using fastp (version 0.23.4) (PMID:30423086) and were aligned to the mouse reference genome (GRCm39) using Hisat2 (version 2.2.1) (PMID:31375807). Then, raw read counts were calculated using feature Counts (version 2.0.5) (PMID:24227677), normalized to CPM values, and used as a measure to quantify the expression abundance of transcripts. The R Language edgeR (version 3.19) (PMID:19910308) package was used to analyze the

differentially expressed genes (DEGs). The criteria for DEGs were followed as $P < 0.05$ and $|log2FC| > 2.0$. The volcano plots and heatmap of the differentially expressed genes were drawn using GraphPad Prism 8.0 software. Hierarchical clustering was conducted using custom Python code, with read counts normalized by CPM values. Kyoto Encyclopedia of Genes and Genomes (KEGG) pathway analysis was performed using the cluster Profiler package of R (version 4.12.6). A mouse transcriptomic microarray dataset (accession number: GSE46275) using doxorubicin (DOX), a drug known to induce immunogenicity of tumor cells, was converted to mouse Ensembl ID and subjected to principal component analysis (PCA) with our R.E RNA seq dataset. All input parameters of the bioinformatics tools used in this analysis were selected according to the recommended defaults of the tools used.

RT-qPCR analysis for MDA5 and INF-β

Total RNA was isolated from cultured cells using TRIzol reagent (Invitrogen), followed by chloroform extraction. cDNA was synthesized from 5 μg of mRNA using the RNA to cDNA EcoDry Random Hexamer Premix (Takara, Japan). Quantitative real-time PCR was performed using EzAmp™ qPCR 2X Master Mix (Elpis Biotech, Korea) on a StepOnePlus™ real-time PCR system (Applied Biosystems, USA) under the following conditions: 40 cycles of 30 s at 95℃, 30 s at 55℃, and 30 s at 72℃. Relative gene expression levels were quantified using the $2^{-\Delta\Delta CT}$ method and normalized to GAPDH mRNA expression. Primers for each gene are as follows: for MDA5[1], MDA5-F(5`-GGATGTTCTGCGCCAAACTG-3`) and MDA5-R(5`-TCTACGTTCCAGGCCTCTGT-3`);for IFN-β[2], IFNb-F (5`-ATAAGCAGCTCCAGCTCCAA-3`) and IFNb-R (5`-CTGTCTGCTGGTGGAGTTCA-3); for GAPDH, gapdh-F(5`-AGTGTTTCCTCGTCCCGTAG-3` ) and gapdh-R(5`-CTGTGCCGTTGAATTTGC-3`).[3]

Quantification of MDA5 protein

Cells were collected and lysed with RIPA buffer (25 mM Tris, pH 7.6, 150 mM NaCl, 0.1% sodium dodecyl sulfate (SDS), 1% sodium deoxycholate, and 1% Triton X-100) supplemented with protease inhibitor for

30 min on ice. Cell debris was removed by centrifugation at 15,000 × g for 20 min, and the supernatant was collected. Protein concentration was determined using the Pierce BCA Protein Assay Kit (Thermo Scientific, USA) with bovine serum albumin (BSA) as a standard. Equal amounts of protein from each sample were mixed with 5× loading buffer, boiled for 5 min at 95℃, separated by SDS-PAGE, and transferred to a polyvinylidene fluoride (PVDF) membrane (Bio-Rad) using the Trans-Blot® Turbo™ Transfer System (Bio-Rad). Membranes were blocked with Tris-buffered saline with Tween (TBS-T: 20 mM Tris, pH 7.4, 150 mM NaCl, and 0.1% Tween-20) containing 5% nonfat dry milk for 1 h at room temperature. After washing, membranes were incubated overnight at 4℃ with primary antibodies against MDA5 (Cell Signaling, #5321, 1:1000) and β-actin (Cell Signaling, #4967, 1:5000) diluted in TBS-T containing 3% BSA. After incubation, membranes were rinsed in TBS-T and incubated with HRP-conjugated secondary antibodies (Cell Signaling, #7074, 1:3000) for 1 h at room temperature. Blots were developed using Pierce ECL Western Blotting Substrate (Thermo Scientific, USA) and imaged with the FluorChem E system (ProteinSimple, USA).

MDA5 silencing assay

B16F10 cells were plated at a density of 2 × 10^5 cells per well in 6-well plates containing Dulbecco's Modified Eagle Medium (DMEM) supplemented with 10% fetal bovine serum (FBS), and incubated at 37℃ in 5% CO_2. Cells were transfected with 50 nM small interfering RNAs (siRNAs) targeting MDA5 using Lipofectamine 3000 (Invitrogen, #L3000015) according to the manufacturer's instructions. After siRNA transfection, cells were incubated for an additional 24 h, followed by treatment with 20 nM R.E. for 18 h. IFN-β expression and MDA5 protein levels were then measured. The siRNA oligo sequences were as follows: si-MDA5-1[4] (sense: 5-AUUGACAUGAUGCAUCUUCUC-3, antisense: 5-

GAAGAUGCAUCAUGUCAAUAU-3); si-MDA5-2[1] (5-GAACAAUGAUGGUGCACAA-3); and si-control[5] (5-UUCUCCGAACGUGUCACGUTT-3).

In vitro cell cytotoxicity assay

Cell viability was evaluated using the Cell Counting Kit-8 (CCK-8; Dojindo, Kumamoto, Japan). Tumor and normal cells were treated with R.E at varying concentrations for 24 or 48 hours, and absorbance at 450 nm was measured using a microplate reader (BioTek Instruments, Winooski, VT, USA) in comparison to untreated control cells.

Detection of HMGB1 and ATP release

To assess drug-induced immunogenic cell death (ICD) of cancer cells, B16F10 cells (1×10^4 cells per well) were treated with R.E (0.01-0.03 μM) or doxorubicin (0.2-1 μM) for 24 hours. ATP was quantified using the ENLITEN ATP Assay System (Promega, Madison, WI, USA), and HMGB1 release was measured using an ELISA kit (Cusabio, Houston, TX, USA), following the manufacturers' recommendation. ATP concentrations in the samples were estimated using GloMax 20/20 luminometer (Promega, Madison, WI, USA) and HMGB1 concentration in the sample was detected using microplate reader (BioTek Instruments).

In vitro BMDC maturation

Conditioned media from R.E-treated B16F10 cells (1×10^6 cells per well) were used to stimulate BMDCs for 24 hours. Maturation markers (CD80, CD86) were assessed using flow cytometry (BD Biosciences) after staining with anti-CD11c-PE (117308), anti-CD80-BV421 (104726) and anti-CD86-APC (105012) antibodies (BioLegend, San Diego, CA, USA).

IFN-β secretion

B16F10, CT26, NIH3T3 and HacaT cells (4×10^5 cells per well) were treated with R.E for 24 hours, and IFN-β secretion in the supernatants was quantified using the LEGEND MAX Mouse IFN-β ELISA Kit (BioLegend, 439407).

Design and characterization of MN patches

MN patches were fabricated through a film rehydration and micro-molding technique, as previously described19. Briefly, Pluronic F127 (400 mg) and R.E (2 mg) were dissolved in 6 mL of methanol and the solvent was removed by rotary evaporation under reduced pressure to form a thin film in a round-bottom flask. The resulting film was rehydrated in 1 mL of an aqueous solution containing PEG (Pluronic F127/PEG = 7:3, w/w) and homogenized by sonication for 10 minutes. The resulting solution was then filtered through a 0.8 μm membrane. For MN fabrication, the filtered solution was cast into a PDMS mold. The molds were cooled at 4 °C for 30 minutes and subsequently vacuumed for 2 minutes to eliminate air bubbles trapped within the mold cavities. After carefully removing the remaining solution from the surface of the mold, a 100 μL aqueous solution of PVA/PVP (25/30% w/v) was applied as a backing layer. The backing layer was evenly spread over the mold and dried at 60 °C for 2 hours. Once completely dried, the MN patches were removed from the mold.

The morphology of the MN array was observed using a scanning electron microscope (SEM; JEOL, Tokyo, Japan) and a stereomicroscope (Carl Zeiss Microscopy GmbH, Gottingen, Germany). To visualize drug distribution within the MN array, Alexa Fluor 488 (AF488) was incorporated into the Pluronic F127 solution in PBS (pH 7.4) and cast into the PDMS molds following the micro-molding protocol described above. Z-stack images of the fluorescently labeled MN array were obtained using a confocal laser scanning microscope (Leica TCS SP8, Leica Microsystems, Wetzlar, Germany) with a step size of 5 μm. The localization and distribution of AF488 within the needle structure were analyzed using Leica Application Suite X (LAS X) software.

To characterize the NMCs generated by the MN dissolution, the MN was immersed in 1 mL of PBS (pH 7.4) and incubated at room temperature for 30 minutes. The resulting NMCs were analyzed for their dimensions and morphology using dynamic light scattering (DLS; Zetasizer Nano ZS90, Malvern Panalytical, Malvern, UK) and transmission electron microscopy (TEM; JEOL, Tokyo, Japan). For TEM analysis, 10 μL of the NMC solution was placed onto a copper grid, allowed to settle for 2 minutes, and stained with 1% aqueous uranyl acetate (5 μL). The stained grid was washed with PBS, air-dried for 10–15 seconds, and imaged under TEM.

Transdermal drug delivery by MN

The cutaneous drug delivery after dermal application of MN was visualized by using a hydrophobic fluorescent probe (DiD)-loaded MN (DiD@MN). DiD@MN was manually injected to the dorsal skin of C57BL/6 mouse and left in place for 1 hour to ensure MN dissolution and drug delivery. The skin was excised and embedded in OCT compound (Tissue-Tek®, Sakura Finetek, Torrance, CA, USA). The embedded samples were promptly frozen at −80 °C for sectioning. For cryo-sectioning, skin blocks were equilibrated to −20 °C and sectioned into 25 μm thick slices using a cryostat. For fluorescence imaging, the intradermal migration of DiD-loaded nanomicelles (DiD@NMC) was assessed by confocal microscopy (Leica Microsystems). Cell nuclei were counterstained with DAPI (4′,6-diamidino-2-phenylindole) in a mounting solution, and a cover glass was placed over the slides for 10 minutes in the dark to improve structural visualization. The skin penetration of MN and the localization of DiD@NMC in the tissue were analyzed using Leica LAS X software. For histological analysis of skin structure post-MN application, skin sections were fixed in 10% neutral buffered formalin for 24 hours, embedded in paraffin, and sectioned into 5 μm thick slices. These sections were deparaffinized using xylene, rehydrated through a graded ethanol series, and stained with hematoxylin and eosin (H&E). The stained sections were imaged using a light microscope (Axio Imager 2, Carl Zeiss Microscopy GmbH, Gottingen, Germany) to assess MN penetration depth and histological changes in the skin caused by MN application.

In vivo biodistribution of NMCs

The *in vivo* lymphatic trafficking and biodistribution of DiD@NMC generated from the MN was observed in melanoma-bearing mice. Tumor-bearing models were established by subcutaneously injecting 1×10^6 B16F10 melanoma cells into the left flank of the mice. Twelve days post-inoculation, animals with tumor volumes of approximately 80 mm^3 were randomly assigned to treatment groups. DiD@MN were manually applied to the skin over the tumor site for 60 minutes before removal. After treatment, the mice were sacrificed, and major organs, including the tumor, TDLN, heart, liver, spleen, kidney, and lungs, were surgically excised. The fluorescence intensity of each organ was measured using an *in vivo* imaging system (SPECTRAL Lago X, Spectral Instruments Imaging, Tucson, AZ, USA) to evaluate the distribution of DiD@NMC across tissues.

Immunohistochemical analysis of TDLN

To confirm the lymphatic localization of DiD@NMC, the inguinal lymph nodes were excised 24 hours after the application of DiD@MN, embedded in OCT compound, and frozen in liquid nitrogen for 10 seconds. Cryosections were prepared by equilibrating tissue blocks to -20 °C and slicing them into 3 µm thick sections using a cryostat. The sections were transferred onto poly-L-lysine-coated glass slides, fixed in ice-cold acetone for 5 minutes, air-dried, and stored at -20 °C. Before staining, slides were washed with PBS and blocked with 5% fetal bovine serum (FBS) in PBS for 1 hour at room temperature. After blocking, the sections were washed with PBS and incubated overnight at 4 °C with the following primary antibodies: rat anti-mouse CD205 (eBioscience, DEC-205), CD169 (eBioscience, Siglec-1), and F4/80 (eBioscience, 14-4801-82) to detect dendritic cells (DCs) and macrophages. After washing with PBS, the slides were incubated with FITC-conjugated anti-rat IgG secondary antibody (Invitrogen, A18866) for 1 hour at room temperature, followed by two additional PBS washes. Fluorescence imaging was carried out using a confocal microscope (Leica Microsystems). Images were analyzed with LAS X software (Leica

Microsystems) to evaluate the localization of NMCs within the lymph nodes and their co-localization with DCs and macrophages.

Systemic cytokine measurement

Blood samples were collected from tumor-bearing mice treated with R.E via intravenous (IV), intratumoral (IT), or MN administration at predetermined time points (3, 6, 24, and 72 hours post-treatment). The collected blood was centrifuged at 13,000 rpm for 15 minutes to separate the serum, which was then stored at -80 °C until analysis. Serum IL-6 levels were quantified using OptEIA™ Mouse IL-6 ELISA kits (BD Biosciences, 555240) according to the manufacturer's protocol. Cytokine concentrations were determined by measuring absorbance at 450 nm with a microplate reader and comparing the results to a standard curve generated from known concentrations of recombinant IL-6. Results were expressed as mean ± standard deviation (SD) for each time point.

Biochemical and hematological evaluations

To evaluate the systemic toxicity of R.E, five-week-old female C57BL/6 mice were divided into two groups: a control group consisting of healthy mice and a tumor-bearing group established by subcutaneous injection of B16F10 melanoma cells. R.E was administered either via intravenous (IV) injection or transdermal MN application. Blood samples were collected into heparinized tubes immediately after sampling to prevent coagulation. The collected samples were frozen on dry ice and stored at -80 °C until analysis. Plasma was separated, and whole blood analysis was conducted following standard protocols to measure multiple systemic toxicity markers.

Tumor growth suppression

Female C57BL/6 mice (5–6 weeks old) were subcutaneously implanted with 1×10^6 B16F10 melanoma cells. Once tumor volumes reached approximately 80 mm^3, the mice were randomly assigned to five treatment groups: PBS-treated control, R.E administered intratumorally (R.E(IT)), R.E delivered via

microneedle (R.E@MN), anti-PD-1 antibody (aPD-1), and the combination of R.E@MN with aPD-1. aPD-1 was administered intraperitoneally. The amount of R.E loaded into the MN tips was calculated based on the volume fraction of the pyramid-shaped needles. Each 15 × 15 MN array had dimensions of 0.20 × 0.20 × 0.52 mm (L × W × H), resulting in a total volume of 1.575 mm^3 (0.007 mm^3 × 225 needles). Drug loading efficiency was 96.8% and the average amount of R.E delivered per MN array was 9.68 ± 0.34 µg. Treatments were administered at a dose of 0.5 mg kg-1 R.E according to a predetermined schedule. Tumor growth was monitored every two days by measuring tumor dimensions using a Vernier caliper. Tumor volume (V) was calculated using the formula: V = 0.5 × W2 × L, where W is the minor axis and L is the major axis of the tumor.

Flow cytometry of tumor and TDLN immune cells

Tumor and TDLN were excised, immersed in PBS, and dissociated through a 70 µm cell strainer (Falcon, Mexico City, Mexico) to obtain a single-cell suspension. Red blood cells (RBCs) were lysed using RBC lysis buffer, and the remaining cells were collected by centrifugation at 2000 rpm for 5 minutes. To prevent non-specific antibody binding, cells were pre-incubated with anti-mouse CD16/32 (Biolegend, 101320) for 15 minutes. For intracellular marker staining, cells were fixed and permeabilized using the Fixation/Permeabilization Kit (BD Bioscience, 554714) and subsequently stained with the following antibodies: anti-CD4-FITC (BD Biosciences, 553046), anti-CD25-APC (BD Biosciences, 557192), and anti-FoxP3-PE (BD Biosciences, 560408) to analyze T_{regs}. To detect DC maturation, single-cell suspensions were stained with anti-CD11c-PE (Biolegend, 117308), anti-CD80-BV421 (Biolegend, 104726), and anti-CD86-APC (Biolegend, 105012). For surface marker analysis, cells were stained with combinations of fluorochrome-conjugated antibodies, including anti-CD3-APC (Biolegend, 100312), anti-CD8-FITC (Biolegend, 100706), and their respective isotype controls (APC Armenian hamster IgG isotype control, Biolegend, 400912; FITC rat IgG2a, Biolegend, 400506) to analyze cytotoxic T cells. For exhausted T cells, anti-CD279 (PD-1)-PE (Biolegend, 109103) was used. To assess immune cell infiltration in tumor, cells were stained with anti-CD11c-PE (Biolegend, 117308), anti-CD80-FITC (BD Biosciences, 553768), anti-

CD40-APC (Biolegend, 124612), anti-F4/80-PE (Biolegend, 123110), anti-CD86-FITC (BD Biosciences, 553691), and anti-CD206-APC (Invitrogen, 2489287). All stained cells were acquired using a flow cytometer (BD FACSAria Fusion, BD Biosciences, Franklin Lakes, NJ, USA), and data were analyzed using FlowJo software (version 10). Debris and doublets were excluded based on forward and side scatter (FSC/SSC) gating. To measure IFN-β expression from different cells in tumor tissues, cells were stained with anti-CD45-FITC (Biolegend, 103108) to identify immune cells, anti-TRP2-AF488 (Santa Cruz Biotechnology, sc-74439 AF488) to identify melanoma cells, and anti-IFN-β-APC (ASSAYPRO, 32183-05161) to detect intracellular IFN-β levels.

In vivo IFNAR1 monoclonal antibody

To validate the role of type I IFN signaling in R.E-mediated anti-tumor activity, mice were intraperitoneally injected with anti-IFNAR1 monoclonal antibody (clone MAR1-5A3, Bio X Cell, BE0241) at a dose of 1mg per mouse once before treatment initiation. Once tumor volumes reached approximately 80 mm^3, the mice were randomly assigned to three treatment groups: PBS-treated control, R.E encapsulated nanomicelle intratumorally (R.E@NMC), and the R.E@NMC combination with anti-IFNAR1 monoclonal antibody. Treatments were administered at a dose of R.E@NMCs according to a predetermined schedule.

Immunofluorescence analysis of tumor sections

To evaluate T cell infiltration and IFN-β expression in tumor tissues, samples were harvested from drug treated mice, fixed in 10% formalin overnight, embedded in paraffin, and sectioned into 6 μm slices. Sections were deparaffinized, rehydrated, and subjected to antigen retrieval by heating in citrate buffer (pH 6.0) at 95 °C for 20 minutes. After PBS washes, the sections were immersed in a blocking solution (5% FBS in PBS) at room temperature for 1 hour. For staining, primary antibodies used included rabbit anti-TRP2/DCT (Abcam, ab74073), IFN-β polyclonal antibody (Invitrogen, PA5-20390), and rat anti-mouse CD8a-PE (BD Biosciences, 553033). Secondary antibodies included goat anti-rabbit IgG H&L-AF647 (Abcam, ab150079) and goat anti-rabbit IgG H&L-AF488 (Invitrogen, A-11034). Nuclei were stained with

Hoechst 33258 (Invitrogen, H3569). Primary antibody incubations were performed overnight at 4 °C, followed by secondary antibody incubation for 1 hour at room temperature. Fluorescence images were obtained by a confocal microscopy and analyzed using Leica LAS X software (Leica Microsystems).

Analysis of tumor antigen-specific CD8^{+} T cells

To evaluate the effects of R.E treatments on systemic immunity, spleens were harvested from treated tumor-bearing mice and processed into single-cell suspensions. Splenocytes were obtained by gently disrupting the spleens through a 70 μm cell strainer, followed by washing and red blood cell (RBC) lysis using RBC lysis buffer. For antigen-specific T cell restimulation, splenocytes were seeded into a 12-well plate at a density of 1×10^{6} cells per well. Cells were incubated with mouse melanoma antigen gp100 (10 μg mL^{-1}), GolgiPlug containing brefeldin A (1 μL, BD Biosciences, 555029), and a cell stimulation cocktail (Invitrogen, 00-4970) for 12 hours at 37 °C. To observe antigen-induced IFN-γ expression, splenocytes were washed and permeabilized using the Fixation/Permeabilization Kit (BD Bioscience, 554714). Non-specific binding was blocked with anti-mouse CD16/32, and the cells were subsequently stained with the following antibodies: anti-CD3-BV421 (Biolegend, 100228), anti-CD8-BV510 (Biolegend, 100752), and anti-IFN-γ-PE (Biolegend, 163504). Flow cytometry analysis was conducted using the BD FACSAria Fusion system, and the data were processed and analyzed using FlowJo software.

Lung and lymph node metastasis analysis

To observe metastasis to the lungs and lymph nodes, the tissue samples were harvested from the tumor-bearing mice after the treatment with either anti-PD-1 (aPD-1) or R.E@MN combined with aPD-1 for 2 weeks. The excised lung and LN tissues were immediately fixed in 10% buffered formalin at room temperature for 24 hours to preserve their structure. After the fixation, the tissues were dehydrated, embedded in paraffin, and sectioned into 6-μm thick slices. The paraffin-embedded sections were mounted on glass slides, deparaffinized, and stained with hematoxylin and eosin (H&E) to visualize metastatic foci. High-resolution imaging of the stained sections was performed using a slide scanner (Axio Scan.Z1, Carl

Zeiss Microscopy GmbH, Göttingen, Germany). Image processing and analysis were carried out using Zeiss ZEN software (Carl Zeiss).

Statistical analysis

All statistical analyses were conducted using GraphPad Prism software (version 8.01, GraphPad Software, La Jolla, CA, USA). For comparisons of two groups, an unpaired Student's t-test was used. Differences among multiple groups were assessed using one-way analysis of variance (ANOVA), followed by appropriate post-hoc tests for multiple comparisons. Results are expressed as the mean ± standard deviation (SD). Schematic illustrations were created using BioRender.com.

A

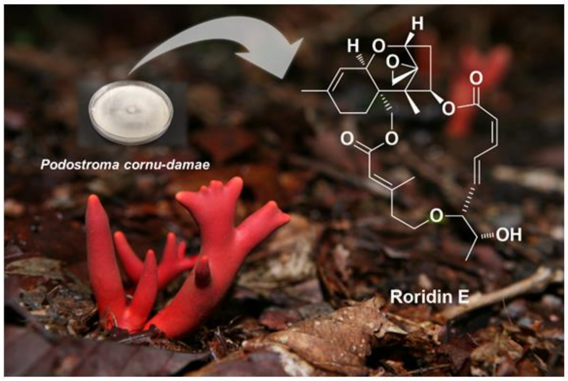

B

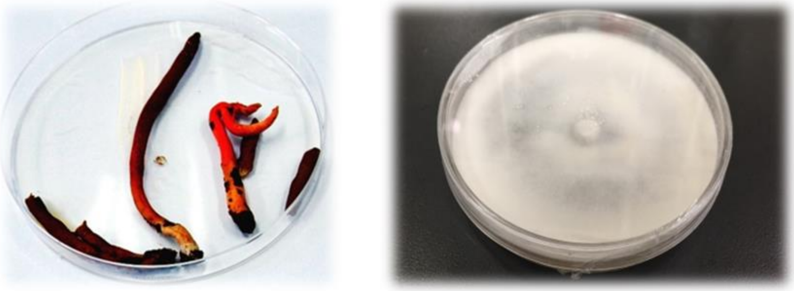

Figure S1. (A) Photograph of *P. cornu-damae* and chemical structure of roridin E. (B) The photo of *P. cornu-damae* (left) and a plate culture of the fungus *P. cornu-damae* (right).

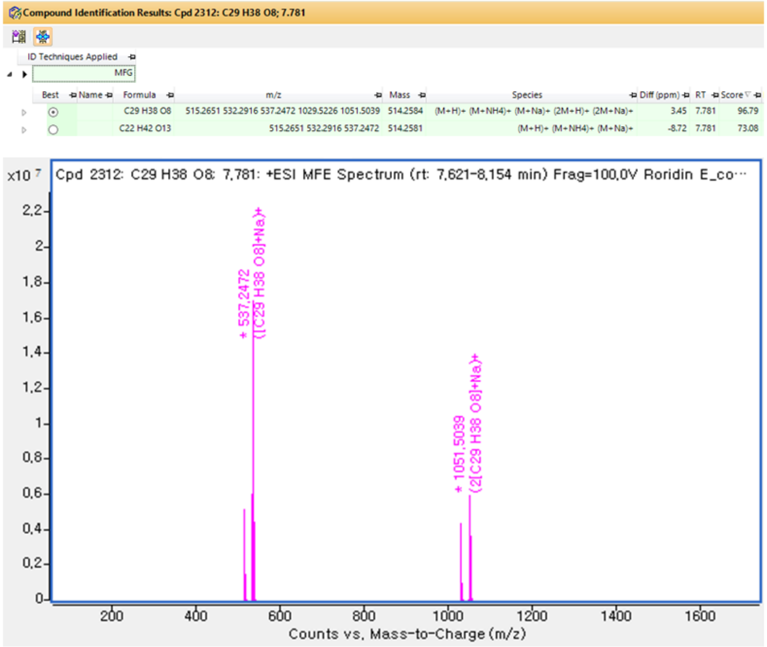

Figure S2. High-resolution electrospray ionization mass spectrometry (HR-ESIMS) data of R.E. HR-ESIMS spectrum of R.E, displaying the detected ions and their corresponding *m/z* values. The data validates the molecular weight and composition of R.E.

A

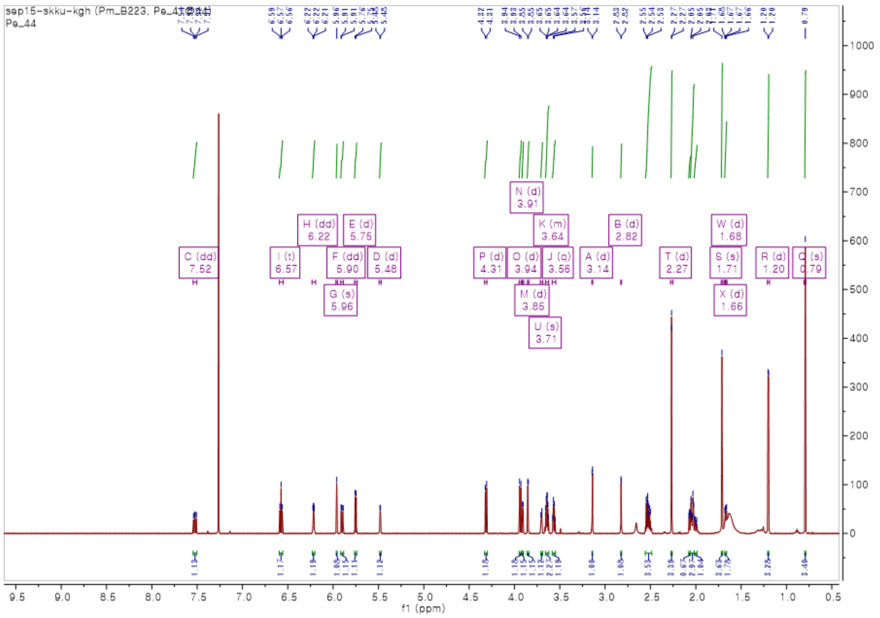

B

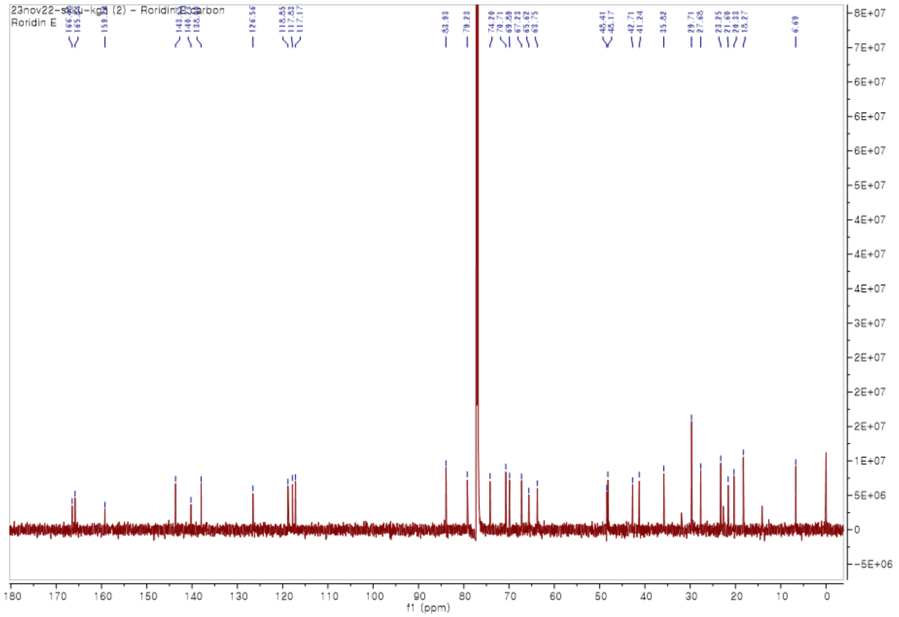

Figure S3. ^{1}H NMR ($CDCl_3$, 850 MHz) and ^{13}C NMR ($CDCl_3$, 212.5 MHz) spectra. (A) ^{1}H NMR (top panel) and (B) ^{13}C NMR (bottom panel) spectra of R.E in $CDCl_3$, recorded at 850 MHz. The spectra display the characteristic proton and carbon signals of R.E, confirming its structural composition.

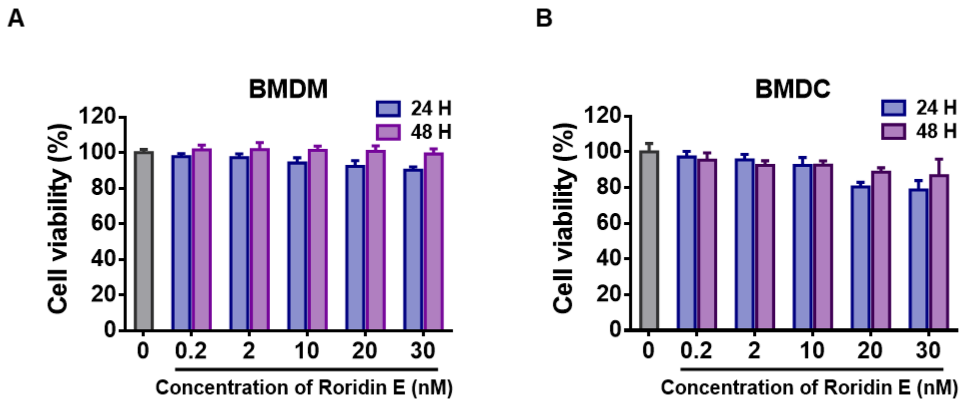

Figure S4. Dose-dependent cytotoxicity of R.E. Dose escalation cytotoxicity assay of R.E on (A**)** BMDM and (B) BMDC for 24 and 48 hours. The results demonstrate the viability of BMDM and BMDC across various R.E concentrations, highlighting the dose-dependent effects over time.

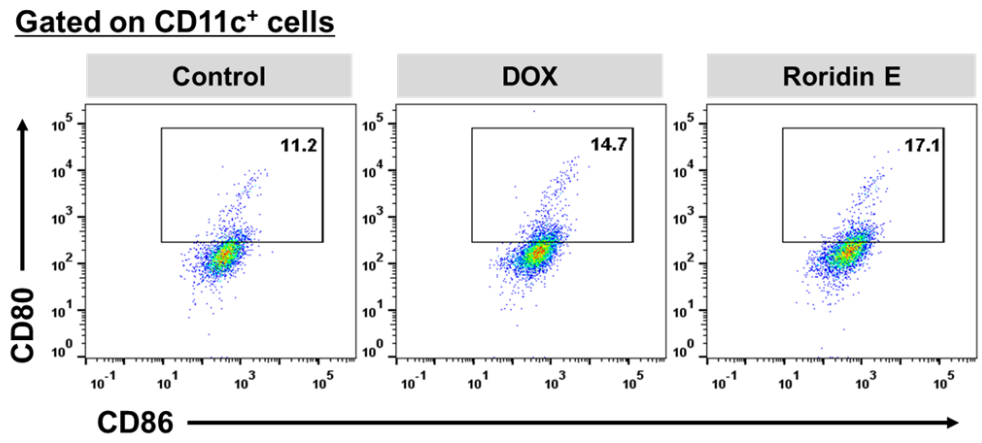

Figure S5. Maturation of BMDCs following 24 hours incubation with R.E. BMDCs were incubated with conditioned medium from roridin E-treated B16F10 cells, and the expression of co-stimulatory markers CD80 and CD86 was assessed. The R.E-treated group shows increased CD80/CD86 expression compared to control and doxorubicin (DOX)-treated groups, indicating enhanced maturation of BMDCs.

A

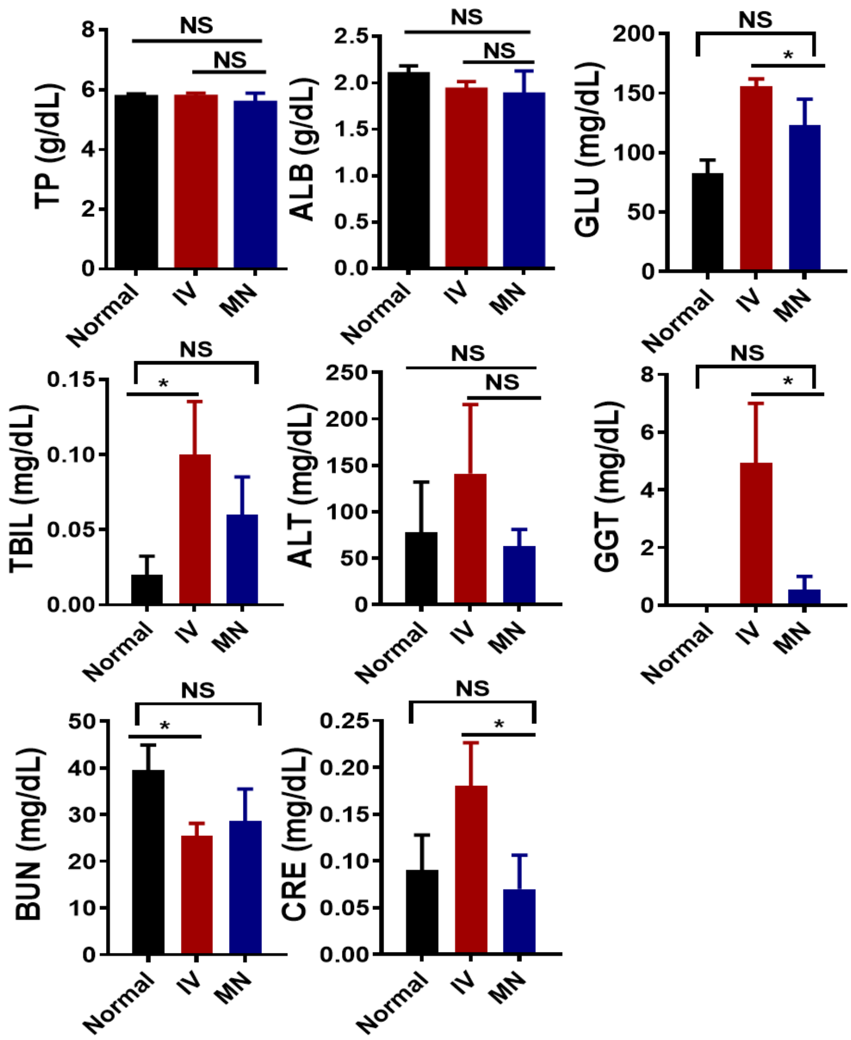

Figure S6. Blood biochemical assay and hematology analysis on day 28 after treatment of R.E in normal mice and tumor-bearing mice. (A) TP, total protein; ALB, albumin; GLU, glucose; TBIL, total bilirubin; ALT, alanine transaminase; GGT, γ-glutamyl transferase; BUN, blood urea nitrogen; CREA, creatinine. (B) WBC, white blood cell; RBC, red blood cell; HGB, hemoglobin; HCT, hematocrit; MCV, mean corpuscular volume; MCH, mean corpuscular hemoglobin; MCHC, mean corpuscular hemoglobin concentration; PLT, platelets. Statistical significance was assessed using Student's *t*-tests for pairwise comparisons and one-way ANOVA for multiple groups. All data are presented as mean ± SD. $^{*}P < 0.05$, $^{**}P < 0.01$, NS = Not significant.

B

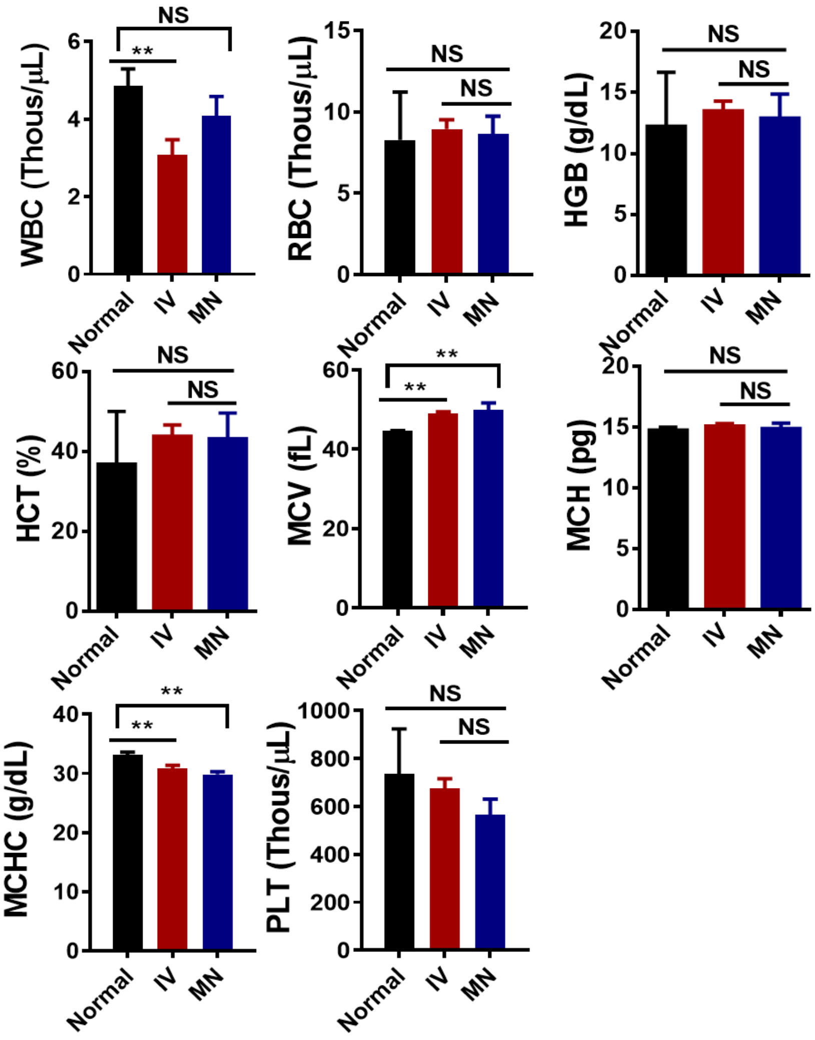

Figure S6. (continued)

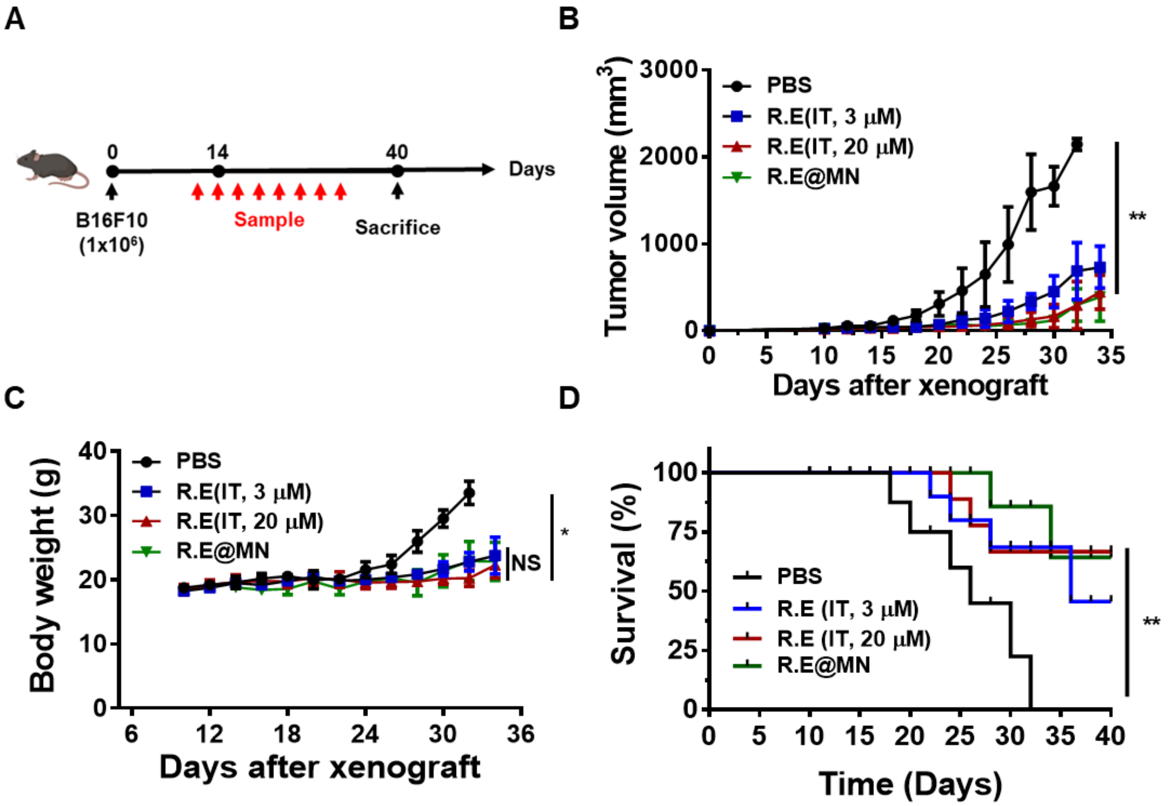

Figure S7. Therapeutic efficacy of R.E in mouse melanoma model. R.E-injected groups inhibit tumor growth and increase overall survival of tumor-bearing mice. (A) Treatment schedule for the primary tumor model, outlining the administration of R.E. (B) Tumor size measurements demonstrating significant tumor growth inhibition in the overall R.E-injected group. (C) Body weight changes in tumor-bearing mice during the treatment period. (D) Survival rate analysis, showing increased overall survival in the R.E-treated group compared to controls. (n = 12-16 per group). Statistical significance was assessed using Student's t-tests for pairwise comparisons and one-way ANOVA for multiple groups. All data are presented as mean ± SD. $^{*}P < 0.05$; $^{**}P < 0.01$, NS = Not significant.

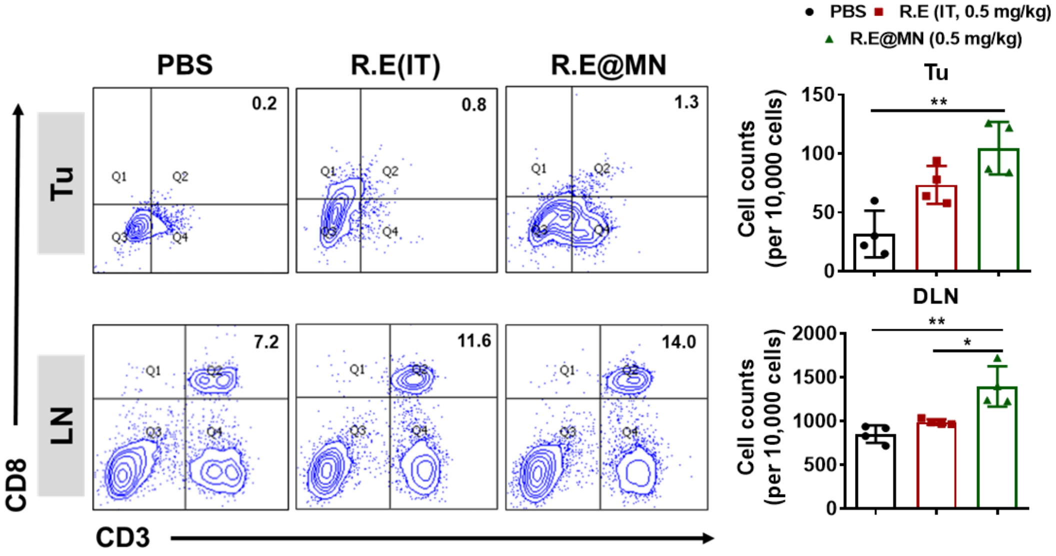

Figure S8. Cytotoxic T cell population in tumor and TDLNs. Flow cytometry analysis showing the percentage of $CD3^+$ $CD8^+$ T cells in tumor tissues and TDLNs of mice treated with PBS, R.E(IT), or R.E@MN in a melanoma model (n = 5 per group). Representative dot plots and quantification graphs indicate increased $CD3^+$ $CD8^+$ T cell infiltration in R.E-treated groups compared to PBS controls. Significant differences were observed, particularly in the R.E@MN group, highlighting its efficacy. Statistical significance was assessed using Student's t-tests for pairwise comparisons and one-way ANOVA for multiple groups. All data are presented as mean ± SD. $^*P < 0.05$; $^{**}P < 0.01$.

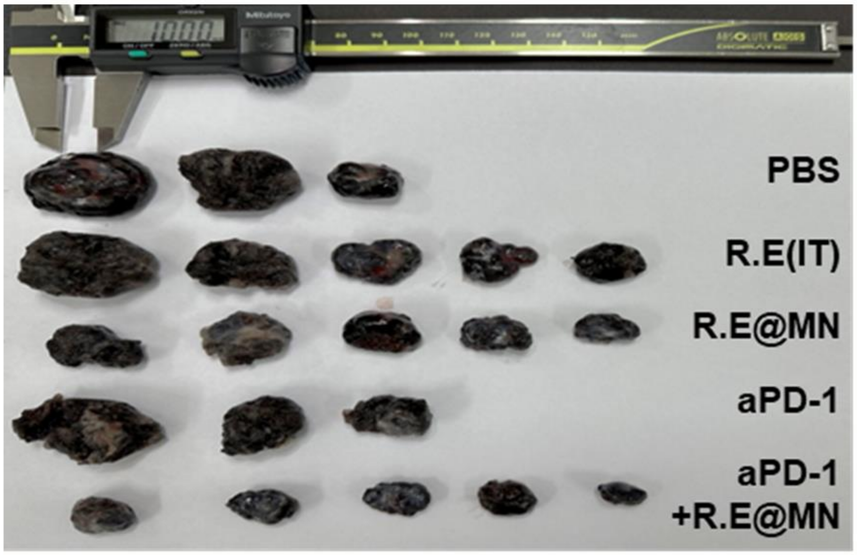

Figure S9. Synergistic anti-tumor effects of R.E and aPD-1 in a B16F10 melanoma model. Representative images of dissected tumors from mice treated with PBS, R.E(IT), R.E@MN, aPD-1 and aPD-1 with R.E@MN. The combination treatment of aPD-1 with R.E@MN shows a pronounced reduction in tumor size compared to single treatments, indicating a synergistic anti-tumor effect.

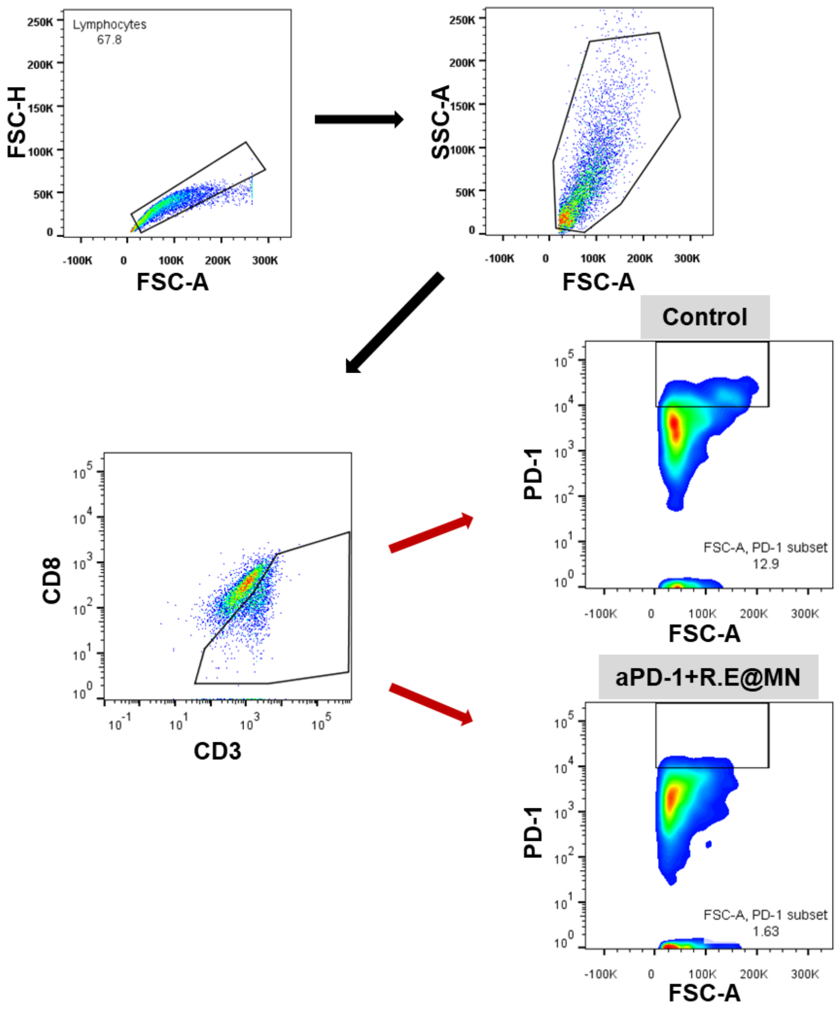

Figure S10. Gating strategy for flow cytometry analysis of exhausted T cells ($CD3^{+}CD8^{+}/PD\text{-}1^{+}$) in C57BL/6 mice. Representative gating strategy for identifying exhausted T cells in the TME of C57BL/6 mice. Flow cytometry analysis was conducted one week after the final treatment to assess the proportion of exhausted T cells. Gating was applied to exclude debris, doublets, and dead cells, followed by the selection of $CD3^{+}CD8^{+}$ T cells and the subsequent identification of $PD\text{-}1^{+}$ subsets within the population.

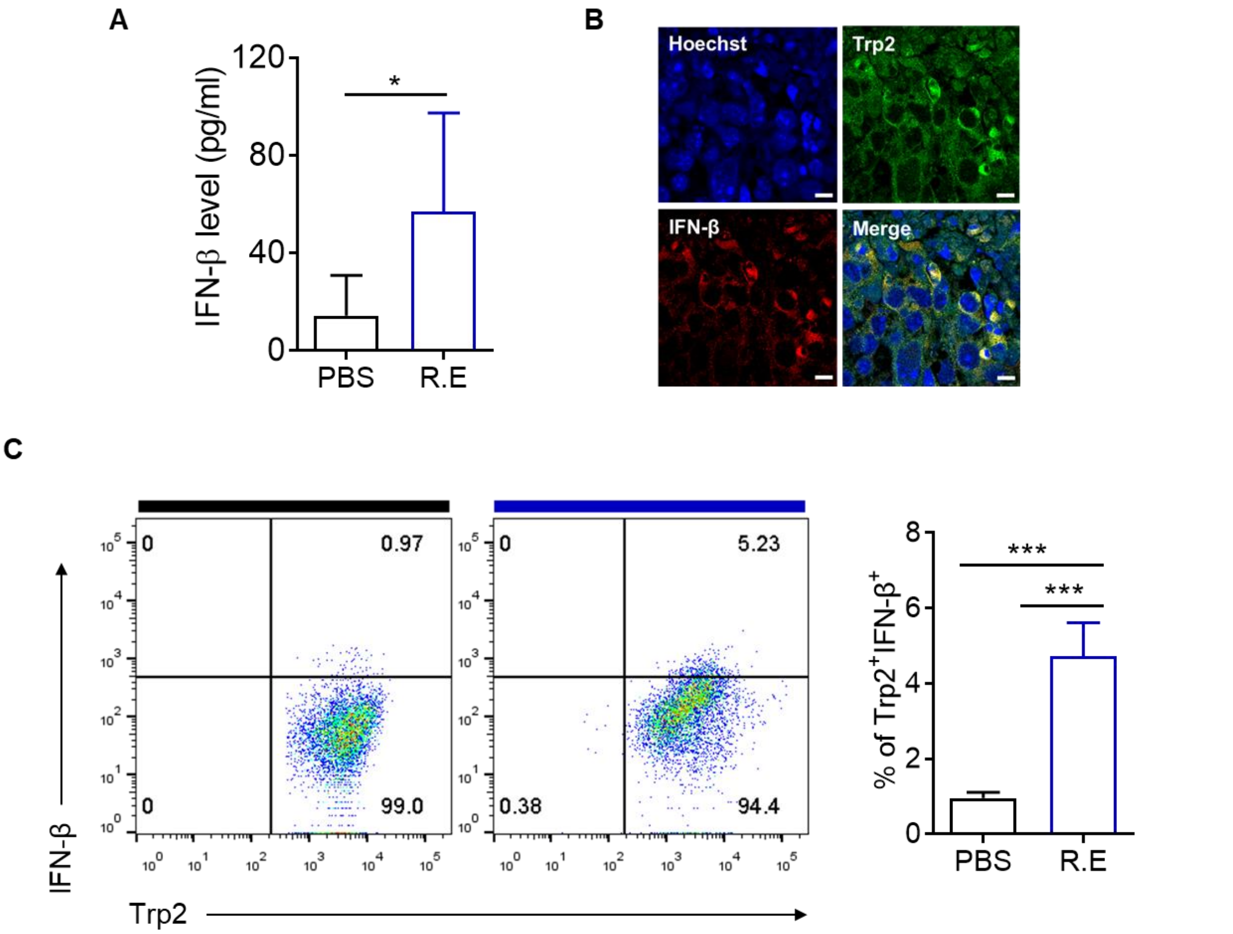

Figure S11. Expression of IFN-β in tumor by R.E. (A) Quantification of the IFN-β production levels in tumors following R.E treatment, indicating enhanced IFN-β release compared to controls. (B) Representative confocal microscopy images of R.E-treated tumor sections, highlighting the accumulation of IFN-β. The slides were stained by Hoechst 33258 (nucleic acid, blue), anti-TRP2/DCT (melanoma, green) and anti- IFN-β (IFN beta, red). (C) Flow cytometry analysis showing the percentage of Trp2^{+} melanoma cells expressing IFN-β. Statistical significance was assessed using Student's t-tests for pairwise comparisons. All data are presented as mean ± SD. $^{*}P < 0.05$, $^{***}P < 0.001$.

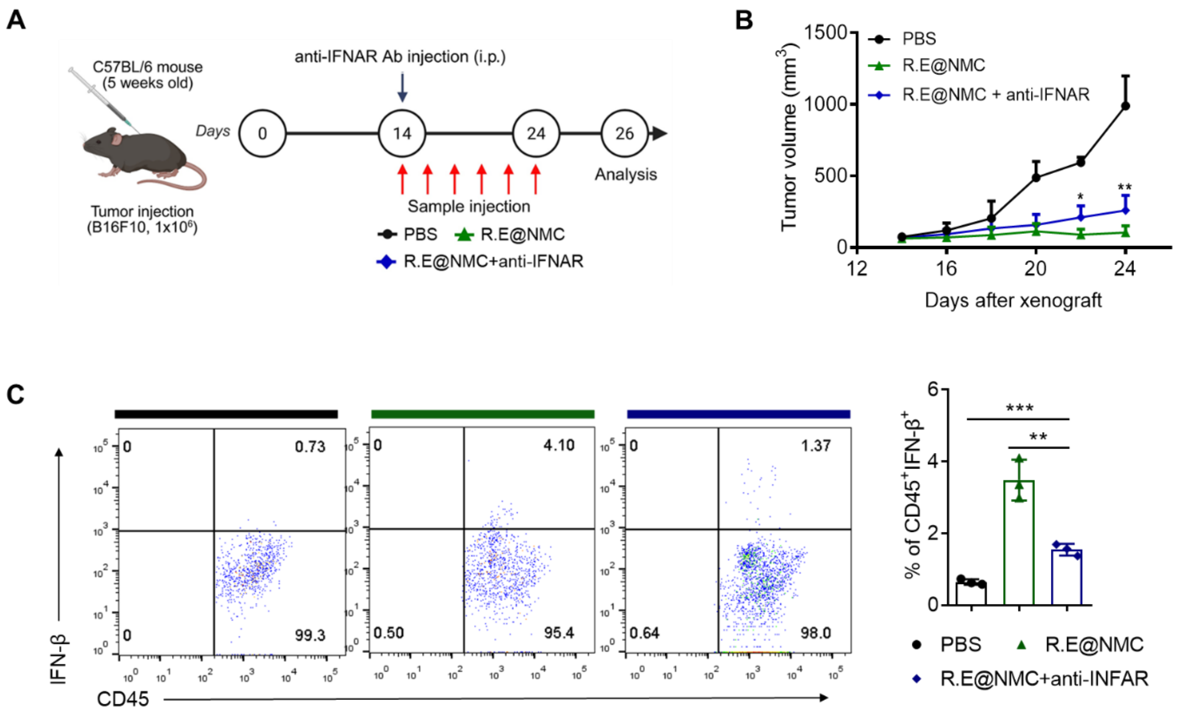

Figure S12. R.E suppresses tumor growth via type-I interferon (IFN-I) signaling. (A) Schematic of the *in vivo* experimental schedule. C57BL/6 mice were subcutaneously inoculated with B16F10 melanoma cells on day 0. A single dose of anti-IFNAR antibody (200 µg, i.p.) was administered on day 14, followed by 2 days interval administration of R.E@NMC. Tumors were collected and analyzed on day 26. (B) Tumor growth during the treatment period. R.E@NMC significantly inhibited tumor growth, whereas anti-IFNAR antibody treatment partially reduced its antitumor efficacy. (C) Cellular analysis of IFN-β expression by $CD45^+$ immune cells in tumor tissues. Tumors were collected and the cells were analyzed by flow cytometry on day 26. Statistical significance was assessed using two-way ANOVA with multiple comparisons test. All data are presented as mean ± SD. $^*P < 0.05$, $^{**}P < 0.01$.

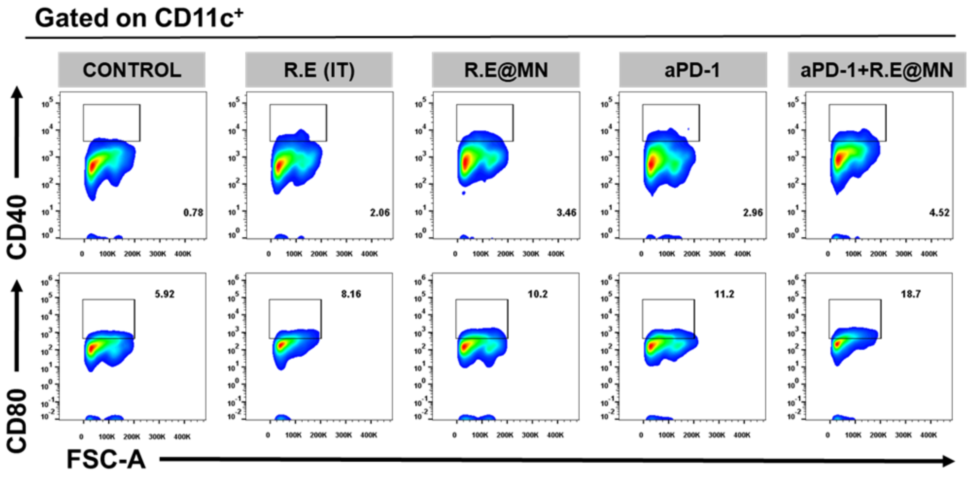

Figure S13. Maturation of recruited DCs within the TME. The intratumoral expression levels of CD40 and CD80 were evaluated from mice treated with PBS (control), R.E(IT), R.E@MN, aPD-1 and aPD-1 with R.E@MN treatment ($n = 4$ per group). Elevated CD40 and CD80 expression in R.E-treated groups, particularly in the combination of aPD-1 with R.E@MN treated group, indicate enhanced DC maturation, which facilitates improved antigen presentation and immune activation within the TME.

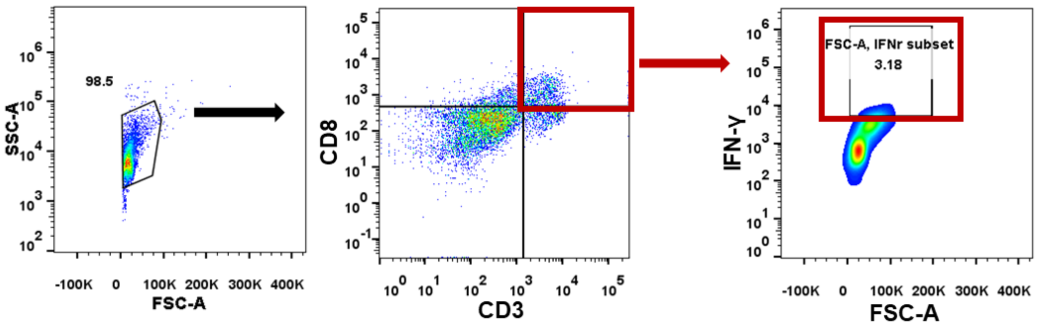

Figure S14. Gating strategy for the determination of T cell subsets in the spleen. Flow cytometry gating strategy used to identify IFN-γ-producing CD8^{+} T cells in the spleen. From the total lymphocyte population, CD3^{+} and CD8^{+} are selected. Within the CD3^{+}CD8^{+} T cell population, IFN-γ expression was used to identify IFN-γ^{+} subsets. The final gate represents the percentage of IFN-γ^{+} CD8^{+} T cells in the spleen.

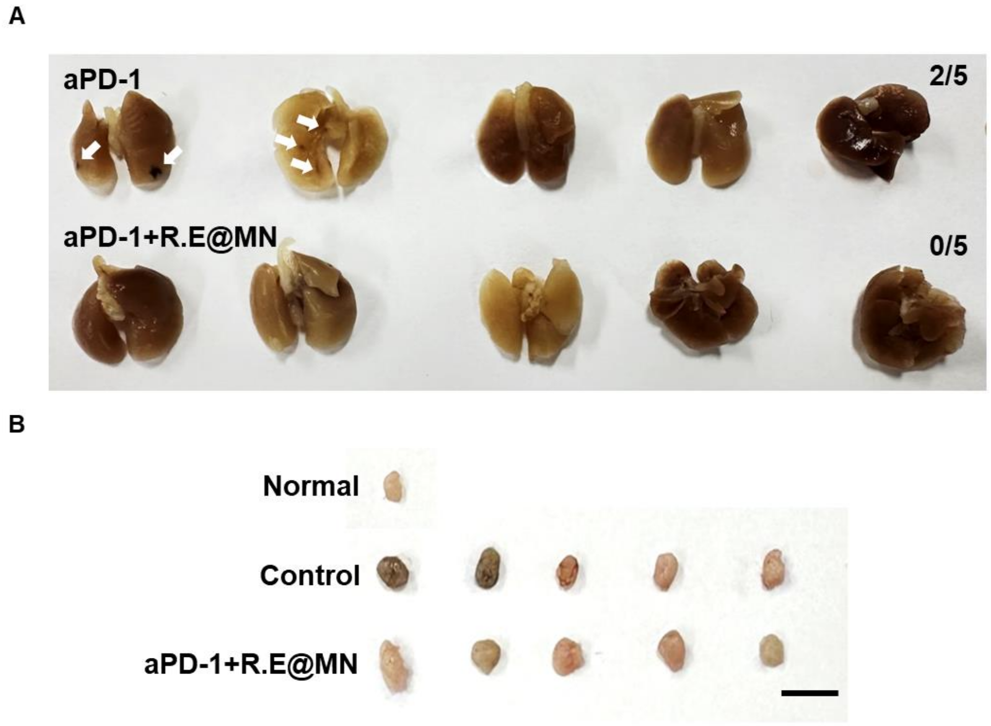

Figure S15. Representative lung and lymph node tissues sectioned from mice. The photographs of representative (A) lung and (B) lymph node tissues isolated from the mice. White arrows indicate visible metastatic nodules in lung tissues, with the number of mice exhibiting metastases shown on the right. Scale bar, 500 μm.

REFERENCES

(1) Shao, Y.; Li, Y.; Liu, Y.; Zhu, S.; Wu, J.; Ma, K.; Li, G.; Huang, S.; Wen, H.; Zhang, C.; et al. ATF3 coordinates the survival and proliferation of cardiac macrophages and protects against ischemia-reperfusion injury. Nat Cardiovasc Res 2024, 3 (1), 28-45. DOI: 10.1038/s44161-023-00392-x From NLM Medline.

(2) Barbalat, R.; Lau, L.; Locksley, R. M.; Barton, G. M. Toll-like receptor 2 on inflammatory monocytes induces type I interferon in response to viral but not bacterial ligands. Nat Immunol 2009, 10 (11), 1200-1207. DOI: 10.1038/ni.1792 From NLM Medline.

(3) Cui, L.; Wang, H. Y.; Ji, Y. X.; Yang, J.; Xu, S.; Huang, X. Y.; Wang, Z. D.; Qin, L.; Tien, P.; Zhou, X.; et al. The Nucleocapsid Protein of Coronaviruses Acts as a Viral Suppressor of RNA Silencing in Mammalian Cells. J Virol 2015, 89 (17), 9029-9043. DOI: 10.1128/Jvi.01331-15.

(4) Nikonov, A.; Molder, T.; Sikut, R.; Kiiver, K.; Mannik, A.; Toots, U.; Lulla, A.; Lulla, V.; Utt, A.; Merits, A.; et al. RIG-I and MDA-5 detection of viral RNA-dependent RNA polymerase activity restricts positive-strand RNA virus replication. PLoS Pathog 2013, 9 (9), e1003610. DOI: 10.1371/journal.ppat.1003610 From NLM Medline.

(5) Geng, S.; Lv, X.; Zheng, W. W.; Xu, T. J. An arms race between 5'ppp-RNA virus and its alternative recognition receptor MDA5 in RIG-I-lost teleost fish. Elife 2024, 13. DOI: ARTN RP9489810.7554/eLife.94898.
